# Two Worlds on a Stone: Arctic Desert Hypoliths and Epiliths Show Spatial Niche Differentiation

**DOI:** 10.1111/gbi.70025

**Published:** 2025-06-26

**Authors:** Andrew Baker, Dale Stokes, Anushree Srivastava, Shannon Rupert, Charles S. Cockell

**Affiliations:** ^1^ University of Manchester Manchester UK; ^2^ Scripps Institution of Oceanography San Diego California USA; ^3^ Carnegie Institute of Science Washington DC USA; ^4^ Mars Society San Diego California USA; ^5^ University of Edinburgh Edinburgh UK

## Abstract

In Arctic polar deserts, rocks can be extensively colonized by phototrophic hypolithic communities that exploit periglacial sorting processes to grow beneath opaque rocks. These communities are distinguished by green bands that are distinctly and abruptly separated from the black‐pigmented communities on the rock surface (epiliths). We used 16S and 18S rDNA culture‐independent methods to address the hypothesis that the two communities are different. Although both communities were dominated by cyanobacterial species (*Chroococcidiopsis* and *Nostoc* spp.), we found that the hypolithic and epilithic habitats host distinct microbial communities. We found that eukaryotic hypolithic and epilithic communities were statistically similar but that the hypolithic habitats contained tardigrade DNA, showing that the more clement subsurface habitat supports animal life in contrast to the surface of the rocks. These results reveal the distinctive communities and sharp demarcations that can develop across small spatial scales in the Earth's rocky extreme environments.

## 1 Introduction

In extreme environments, microorganisms can find refuge on the underside of rocks, the “hypolithic” habitat. The hypolithic habitat has attracted particular interest in extreme environments such as hot and cold deserts because it can provide a more clement environment supporting microbial growth compared to the more exposed upper surface of rocks, making it one of the few habitable locations for life in such settings (e.g., Berner and Evenari 1978; Broady 1981; Budel and Wessels 1991; Chan et al. 2012; Cowan et al. 2011; Gwizdala et al. 2021; Pointing et al. 2007, 2009; Schlesinger et al. 2003; Smith et al. 2014, 2000; Warren‐Rhodes et al. 2006, 2007, 2013; Tracy et al. 2010; Wong et al. 2010). The underside of rocks can offer several advantages for microbial growth, including the trapping of water, screening of UV (ultraviolet) radiation by the overlying substrate, and amelioration of temperature extremes (Cowan et al. 2011; Chan et al. 2012; McKay 2016).

The microbial communities in the hypolithic habitat have been found to be distinct from the surrounding soils (Makhalanyane, Valverde, Birkeland, et al. 2013; Makhalanyane, Valverde, Lacap, et al. 2013; Wei et al. 2016), with hypolithic communities displaying high diversity in metabolic pathways to cope with nutrient limitation (Wei et al. 2016).

Photosynthetic microorganisms in hypolithic habitats have been typically associated with quartzitic rocks because of the translucency of these substrates, which permits photosynthetically active radiation (PAR) to penetrate to the underside of the rock. In contrast, extensive colonization of the underside of opaque rocks has been reported in the Antarctic and Arctic by photosynthetic microorganisms (Cockell and Stokes 2004, 2006). In polar environments, periglacial processes, such as freeze–thaw cycles, cause rocks to sort and move into characteristic geological formations such as polygonal patterned ground (Kessler and Werner 2003). As rocks move and assemble into these formations, light can penetrate around the rock edges to the underside where there is sufficient separation between soil and rock, explaining how photosynthetic microbes can colonize the underside of opaque rocks. In these patterned grounds, hypolithic communities form distinct bands of green on the rock underside. These hypoliths can contribute significantly to carbon fixation in environments with limited surface plant cover (Cockell and Stokes 2004), potentially fixing as much carbon as the above‐ground biomass.

While polar hypolithic communities experience better growth conditions compared to the surface, for example by protection from UV radiation and access to snowmelt that tends to accumulate there, the surfaces of numerous polar rocks are also colonized by a conspicuous black layer of microbial growth. This creates a well‐defined boundary between the black (surface epilithic) and green (hypolithic) layers.

There are two possible hypotheses to explain this phenomenon. One hypothesis is that the hypolithic and epilithic communities are identical, with the epilithic microorganisms producing black pigmentation as a response to higher UV radiation exposure on the surface of the rock compared to the underside. An alternative hypothesis is that the microorganisms present on the exposed surface and underneath the rock represent two distinct communities that have developed in response to varying physical extremes (however, in this scenario, it is still possible that the dark surface pigmentation on the rock surface is a response to UV radiation in some of the same microorganisms found on the underside).

The aim of our study was to test the hypothesis that the hypolithic and epilithic microbial communities of Arctic rocks are different and that the visual sharp demarcation of the pigmented communities on the surface and underside of these rocks is reflected in their community composition.

## 2 Methods

### 2.1 Sample Collection

**FIGURE 1:**
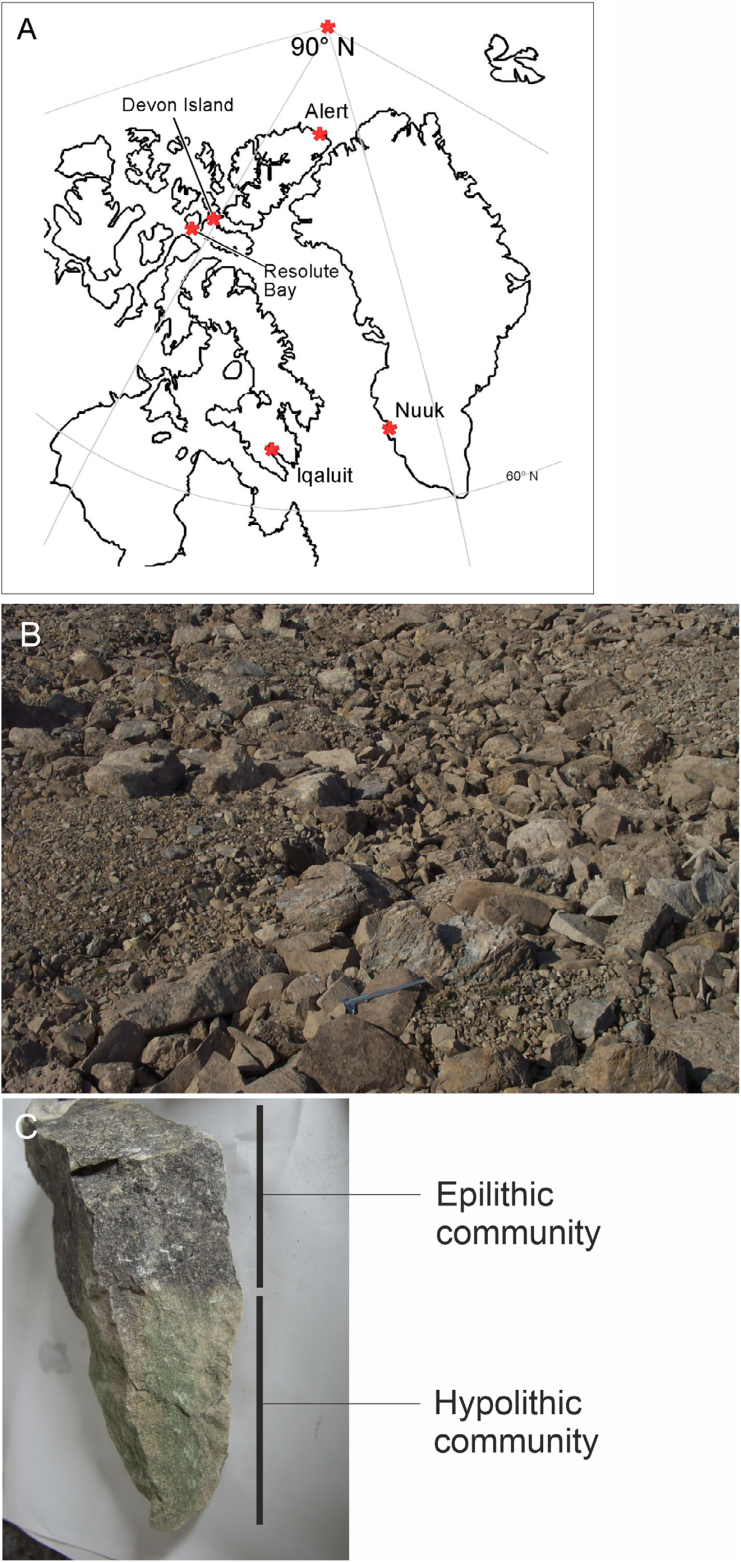
Hypolithic communities of Devon Island, Canadian High Arctic. (A) Location of sampling site in Canadian High Arctic (Devon Island), (B) An example of patterned ground on Devon Island showing sorting of rocks and edges around which light can penetrate to the subsurface. The image is approximately two metres across in the foreground, (C) An example of a rock community inhabiting Arctic rocks. The hypolithic community is shown as a distinctive green region on the underside of the rock. The surface of the rock is covered by a black‐pigmented epilithic community. The rock is 21 cm tall.

Samples of rocks were obtained from the Haughton Impact Structure on Devon Island, in the Canadian High Arctic (Figure 1). Rocks were selected in patterned ground that displayed clearly defined green (underside; hypolithic) and black pigmented (surface; epilithic) communities (Cockell and Stokes 2004, 2006). Rocks were dried and maintained in a desiccated state until returned to the laboratory. They were kept in enclosed containers to minimize contamination from the environment. All rock substrates were limestone.

Samples were examined from six distinct locations: (1) 75°25.415′ N; 89°50. 63′ W; (2) 75°25.450′ N; 89°50.446′ W; (3) 75°26.125′ N; 89°49.401′ W; (4) 75°26.227′ N; 89°49.578′ W; (5) 75°26.028′ N; 89°46.989′ W; (6) 75°25.447′ N; 89°50.435′ W. Samples were collected using a 1 m^2^ quadrat grid, and all rocks were visually examined (colonized or uncolonized) to select colonized rocks. Rocks of ≥ 5 cm length were sampled in which the size of the rock was generally sufficient to allow for the development of distinguishable epilithic and hypolithic biofilm growth. Three rocks from each location were processed. For each rock, sterile scalpels were used to separately scrape the hypolithic (the green subsurface biofilm of the rock) and epilithic (the black surface biofilm of the rock) into sterile Petri dishes in a laminar flow hood, observing aseptic technique, removing as much of the community as possible from the rock, which included material from the complete community up to the transition zone between the hypolithic and epilithic community. The three samples from each location were pooled to generate one sample for each location (a total of six samples).

### 2.2 Extraction of DNA

The dry scrapings from the hypolithic and epilithic communities were used for DNA extraction. For all samples, approximately 250 μg of material was added to extraction buffer using the Qiagen DNeasy PowerSoil Kit (Qiagen, Hilden, Germany) and DNA was extracted according to the manufacturer's instructions. DNA samples were sent for analysis to Research and Testing Genomics Laboratories (RTL, Lubbock, TX, USA). Bacterial sequences were examined using 16S rDNA primers (28F [GAGTTTGATCNTGGCTCAG]‐388R [TGCTGCCTCCCGTAGGAGT]). A cyanobacterial specific set of primers was used to examine this phylum in the 16S RNA region (359F [GGGGAATYTTCCGCAATGGG]‐781R [GACTACWGGGGTATCTAATCCCWTT]). The eukaryotic component of the community was examined using 18S rDNA primers for the V4 region (TAReukF [CCAGCASCYGCGGTAATTCC]‐TAReukR [ACTTTCGTTCTTGATYRA]).

### 2.3 DNA Analysis

After receiving data from RTL, the data were processed as follows. RTL returned a database of ordinary taxonomic units (OTU's) which gave classifications to the best available taxonomic resolution, which utilises the NCBI/EMBL/DDBJ database (RTL Genomic, 2019). In some cases, the taxonomic information is reported as “Unknown,” where RTL's algorithm was unable to make a confident determination at that taxonomic level, or “Unclassified,” where NCBI contains missing information at that taxonomic level.

The database was first trimmed to remove all “species” which RTL had been unable to identify to at least class level, including both “Unknown” and “Unclassified” data, so as to negate the statistical effect of any species which were unidentifiable, and thus could not provide any important information about the composition of the communities (RTL Genomics 2019). The trimmed dataset was then prepared for statistical analysis in PRIMER. Data was received in PRIMER as an “Abundance” dataset, taken from the number of identified sequences for each OTU within each habitat. The dataset was then split into two factors, representing samples from the hypolithic and epilithic communities. A square root transformation was applied separately to the prokaryotic and eukaryotic communities and following this transformation, a similarity matrix was produced for each dataset using the Bray‐Curtis resemblance measure (Beals 1984). The two matrices produced were then subjected to statistical testing.

### 2.4 Statistical Analysis

Multivariate analysis can provide a holistic view of an ecosystem, and highlight the differences between two communities. Here, two tests were applied. The first, an Analysis of Similarity, or ANOSIM test (Clarke 1993) where the relative “distance” (or similarity) between samples of a particular group are compared to the relative distance between samples of different groups to produce an “*R*” value; a high *R* value indicates that samples are more similar to samples within their own group, and thus there is a likely difference in community composition or relative abundance between the two groups. The second, a Similarity Percentages, or SIMPER test, was applied to the original datasets (post‐transformation) (Clarke 1993). SIMPER attempts to quantify the relative contribution of different species to the similarity found within groups, and their relative contribution to the differences between groups. Thus, SIMPER analysis provided a list of important indicator species for each environment, which could be used to characterise that community and define the differentiator species.

In addition to these tests, univariate statistical analysis was carried out on the bacterial dataset (post‐transformation) to supplement the multivariate results. Four different univariate statistical indices were examined. The number of species (*S*) and the number of individuals (*N*) provide basic information about an ecosystem and are combined into a Margalef species richness index (*d*) (Margalef 1958), which is defined as:

$$d=\frac{S-1}{\ln N}$$

This index aims to reduce the effects of sampling size and effort, and can provide a more robust indication of community structure. Finally, we assessed the evenness of species using Pielou's evenness index (*J*) (Pielou 1975). This is defined as:

$$J=\frac{H^{\prime }}{\ln S}$$

Here *H*′ represents the Shannon‐Wiener diversity index (Nolan and Callahan 2006), which itself is defined as:

$$H^{\prime }=\sum_{i=1}^{S}P_{i}\ln P_{i}$$

In this case, *S* again refers to the total number of species, and *P*_*i*_ refers to the proportion of individuals belonging to the *i*th species. This was used in the Pielou's index to describe the distribution of individuals across species in a community. If *J* = 1, then all species are equally represented, with an even number of individuals of each taxa. Pielou's evenness index approaches zero as one species begins to dominate the community.

### 2.5 Phylogenetic Analysis

Using the lists of characteristic species provided by SIMPER analysis, we were able to construct comprehensive phylogenetic trees for both communities, including prokaryotic and eukaryotic species. These trees were produced using phyloT v2 and subsequently edited using ITOL (Interactive Tree of Life) (Letunic and Bork 2021).

## 3 Results

### 3.1 Distinct Bacterial Communities Inhabit the Hypolithic and Epilithic Habitats

**FIGURE 2:**
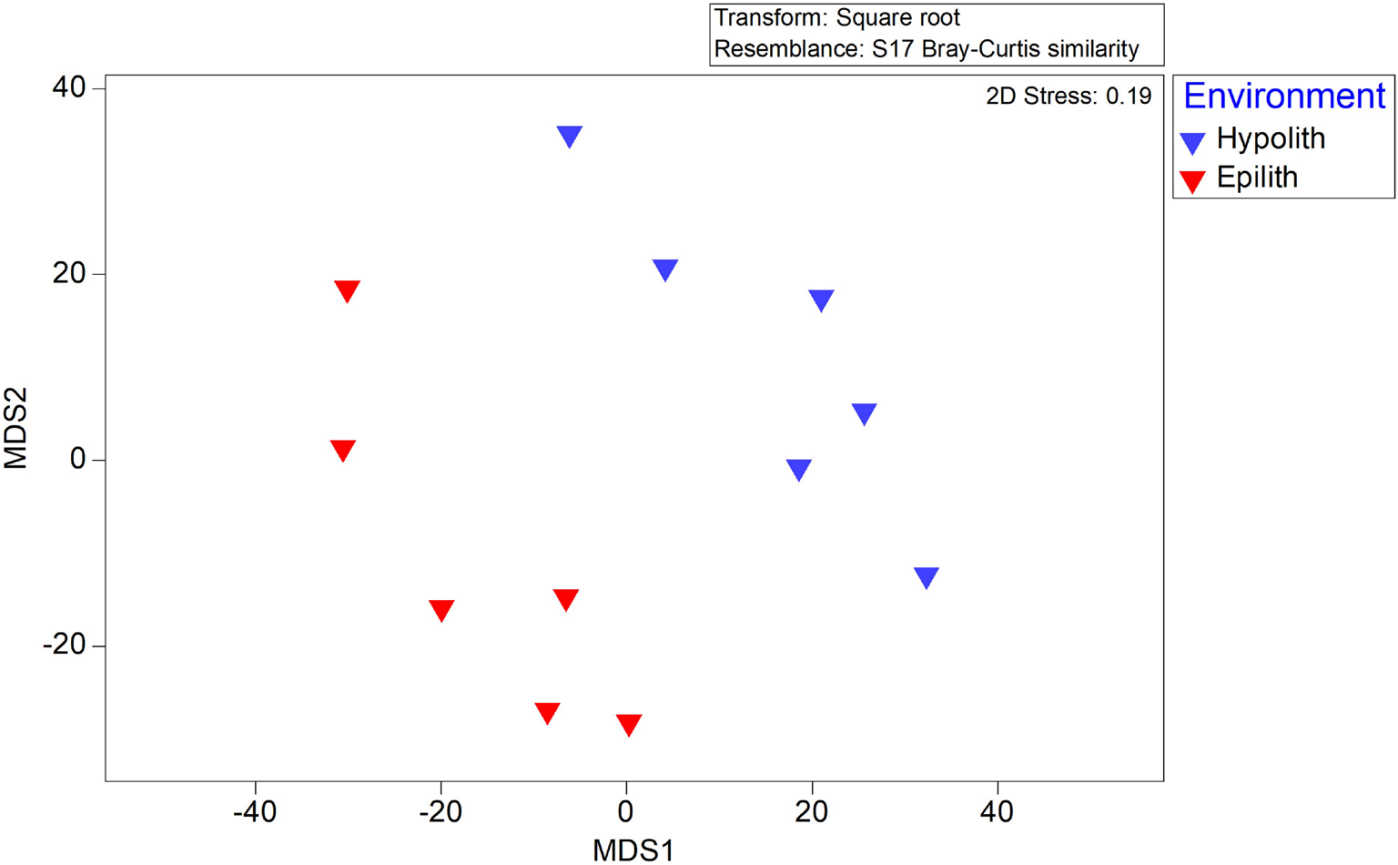
Multi‐Dimensional Scaling plot showing the relative dissimilarity between prokaryotic communities from the hypolithic (blue) and eplithic (red) communities, informed by a Bray–Curtis dissimilarity matrix.

**FIGURE 3:**
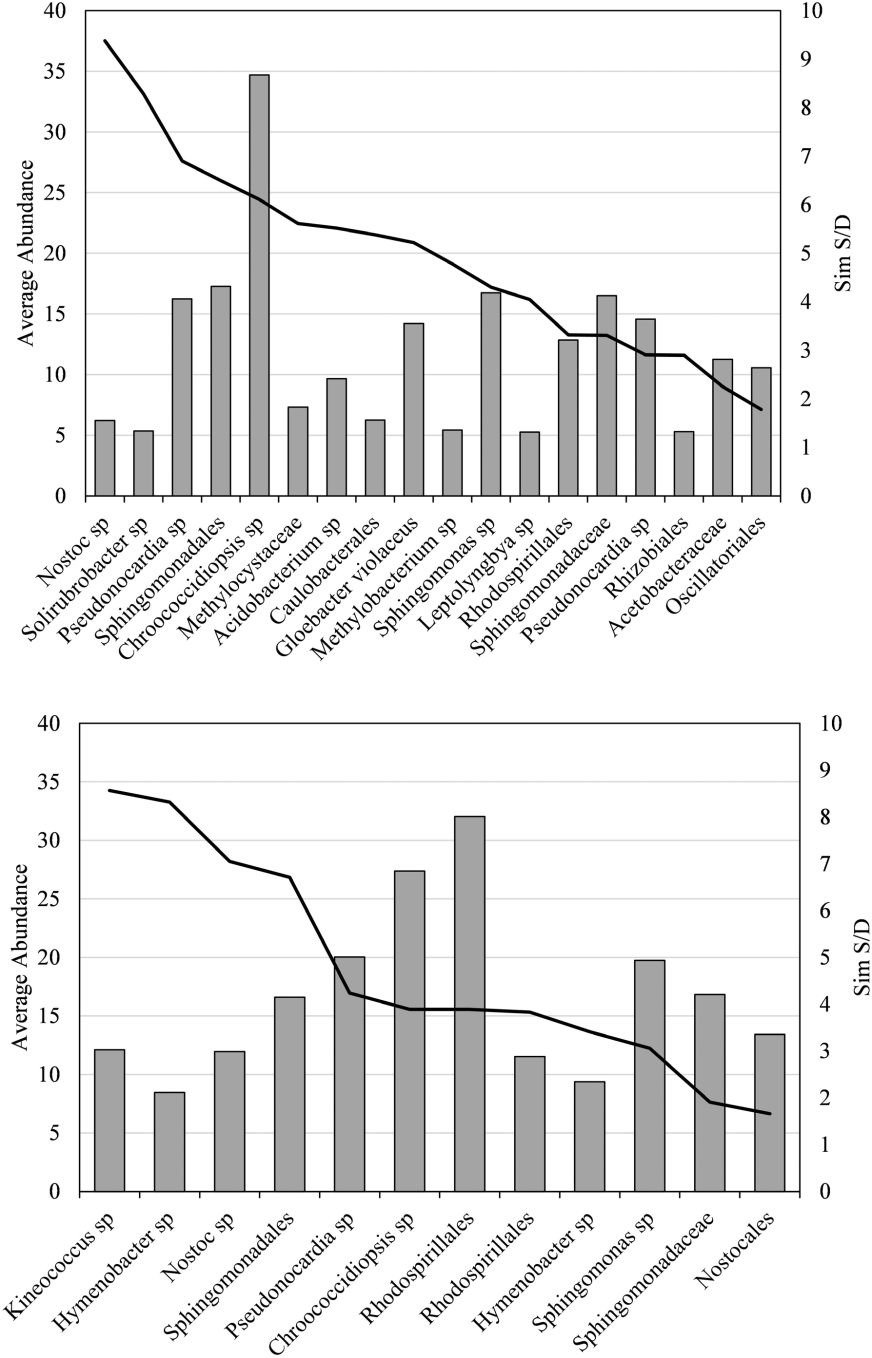
The mean abundance (bars) and statistical importance (line) of each prokaryotic taxa characteristic of hypolithic (upper graph) and epilithic (lower graph) communities. Importance determined by SIMPER analysis.

Phylogenetic analysis showed that distinctive communities inhabit the hypolithic and epilithic habitats. ANOSIM analysis was used to quantify this difference. Analysis of the bacterial communities returned an *R* value of 0.75 (Figure 2) which displays the relative distance (similarity) between communities of each environment, which was found to be highly significant (*p* < 0.01). This indicates a difference between the two communities in terms of community composition and relative abundance of species. SIMPER analysis further revealed the most important indicator species of the hypolithic bacterial community (*n* = 18) as shown in Table 1, Table S1, and of the epilithic bacterial community (*n* = 12) in Table S2. SIMPER also revealed the key bacterial species for differentiating between communities, shown in Table 2, Table S3. Figure 3 shows the average abundance and statistical importance of each indicator species in the hypolithic and epilithic communities determined by SIMPER analysis. These data are also depicted as relative importance of each species in characterizing each community (Figure S1) and the abundance (on a logarithmic scale) of each differentiator species in each community, along with the relative importance of that species in differentiating between the communities (Figure S2).

**TABLE 1:** The top 10 bacterial indicator species associated with the hypolithic community.

Taxon	Phylum	Av. Abundance	Av. Sim	Sim/SD	Contribution (%)	Cumulative contribution (%)
*Chrooococcidiopsis* sp.	Cyanobacteria	34.69	8.17	6.08	12.34	12.34
Sphingomonadales	Pseudomonadota	17.26	3.80	6.61	5.75	18.09
*Sphingomonas* sp.	Pseudomonadota	16.73	3.57	4.38	5.39	23.48
*Pseudonocardia* sp.	Actinobacteria	16.25	3.52	7.10	5.33	28.80
Sphingomonadacaea	Pseudomonadota	16.52	3.32	3.35	5.01	33.81
*Pseudonocardia*	Actinobacteria	14.56	3.21	2.98	4.85	38.66
*Gloeobacter violoceus*	Cyanobacteria	14.21	2.89	5.22	4.37	43.03
Rhodospirillales	Pseudomonadota	12.85	2.44	3.22	3.69	46.72
*Acidobacterium* sp.	Acidobacteriota	9.05	2.15	5.71	3.25	49.97
Acetobacteraceae	Pseudomonadota	11.27	1.99	2.26	3.01	52.98

*Note:* Taxon is assigned to the highest identified level. Information includes the taxon and phylum of each species, as well as the average abundance of sequences (Av. Abundance), calculated average similarity (Av. Sim), similarity/standard deviation ratio (Sim/SD), and the SIMPER calculated contribution of each species to overall similarity, which is also given cumulatively.

**TABLE 2:** The top 10 differentiating bacterial taxa between hypolithic and epilithic community.

Taxon	Phylum	Av. Abundance (hypolith)	Av. Abundance (epilith)	Av. Diss	Diss/SD	Contribution (%)	Cumulative contribution (%)
Rhodospirillales	Pseudomonadota	12.85	32.03	2.61	1.20	5.82	5.62
*Pseudonocardia*	Actinobacteria	14.56	2.28	1.67	2.99	3.74	9.56
*Nostocales*	Cyanobacteria	2.96	13.41	1.50	1.83	3.36	12.92
*Chroococcidiopsis* sp.	Cyanobacteria	34.69	27.38	1.28	1.50	2.86	15.77
Kinecoccus	Actinobacteria	3.93	12.10	1.12	4.45	2.50	19.27
*Gloeobacter violoceus*	Cyanobacteria	14.21	5.34	1.10	1.80	2.47	20.74
*Hymenobacter*	Bacteroidota	1.51	9.37	1.06	1.83	2.38	23.12
*Hymenobacter* sp.	Bacteroidota	1.14	8.45	0.39	2.43	2.21	25.33
*Oscillatoriales*	Cyanobacteria	10.57	3.85	0.37	1.98	2.17	27.50
Sphingomonodaceae	Pseudomonadota	16.52	16.82	0.34	1.28	2.10	29.60

*Note:* Taxon is assigned to highest identified level. Information includes the taxon and phylum of each species, as well as average abundance of sequences in hypolith and epilith environments (Av. Abundance), average dissimilarity (Av. Diss), dissimilarity/standard deviation ratio (Diss/SD), and the SIMPER calculated contribution of each species to overall dissimilarity, which is also given cumulatively.

**FIGURE 4:**
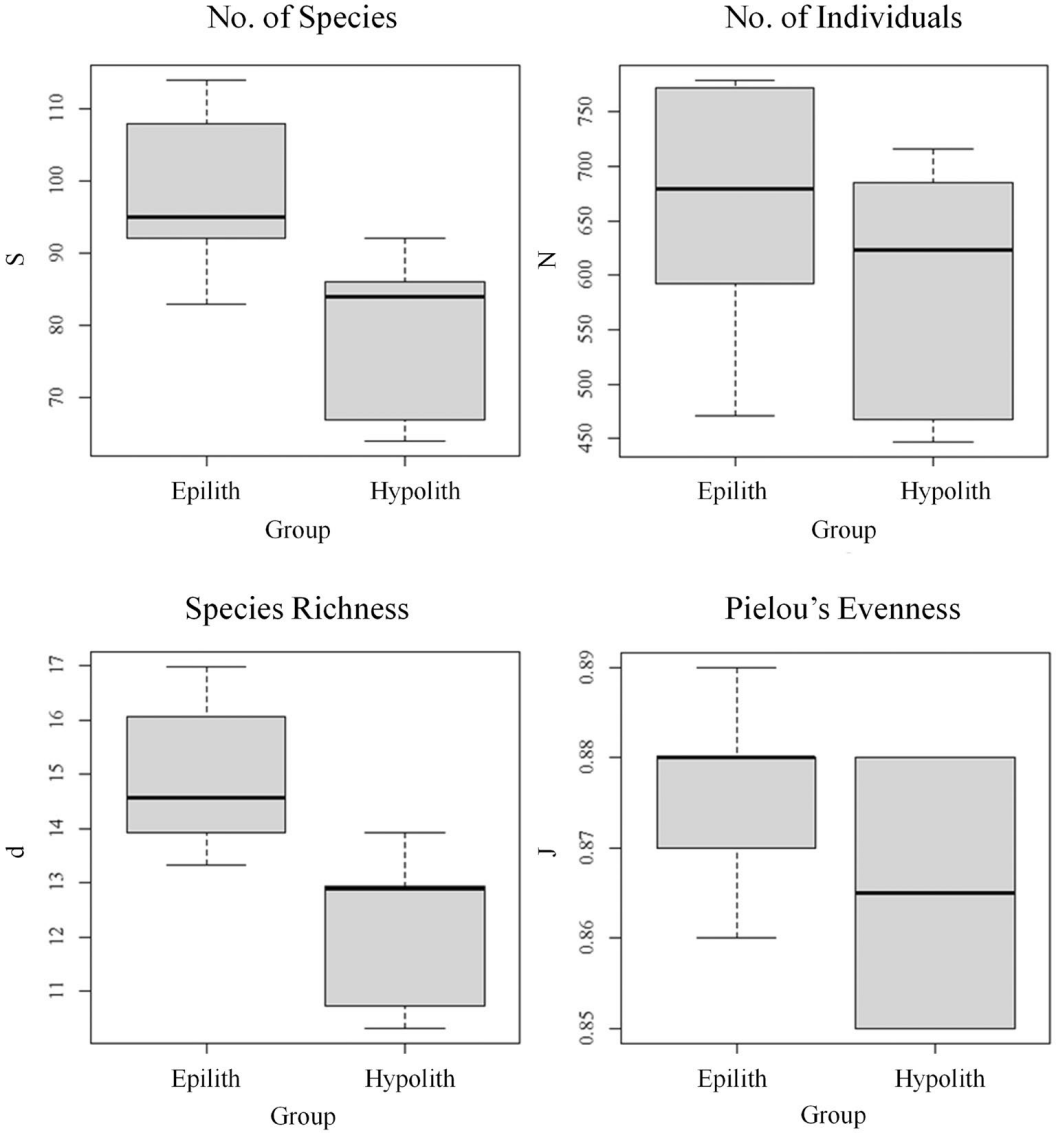
Univariate analysis of prokaryotic communities comparing hypolithic and epilithic communities. Data show the total number of species (*S*), total number of individuals (*N*), species richness (*d*), and Pielou's species evenness (*J*).

The results of the multivariate statistics can be better understood by investigating the univariate statistics of the two communities. The results of the univariate analysis on bacterial communities are shown in Figure 4. The mean *S* scores were hypolithic, 88.67 ± 14.42, and epilithic, 79.50 ± 11.27. Pairwise Welch two‐sample *t*‐tests revealed that the hypolithic and epilithic communities were significantly different from one another (*p* < 0.05).

The mean *N* scores were hypolithic, 627.92 ± 114.75 and epilithic, 593.67 ± 112.21. Pairwise Welch two sample *t*‐tests revealed that the hypolithic and epilithic communities were not significantly different from each other (*p* > 0.05).

The mean *d* scores were hypolithic, 13.60 ± 1.91, and epilithic, 12.29 ± 1.43. Pairwise Welch two‐sample *t*‐tests revealed that the hypolithic and epilithic communities were significantly different from one another (*p* < 0.05).

The mean *J* scores were hypolithic: 0.870 ± 0.013 and epilithic: 0.865 ± 0.014. Pairwise Welch two sample *t*‐tests revealed that the hypolithic and epilithic communities were not statistically different from each other (*p* > 0.0.5).

### 3.2 Eukaryotic Communities Were Not Significantly Different, but the Hypolithic Environment Supported Animal Life

**FIGURE 5:**
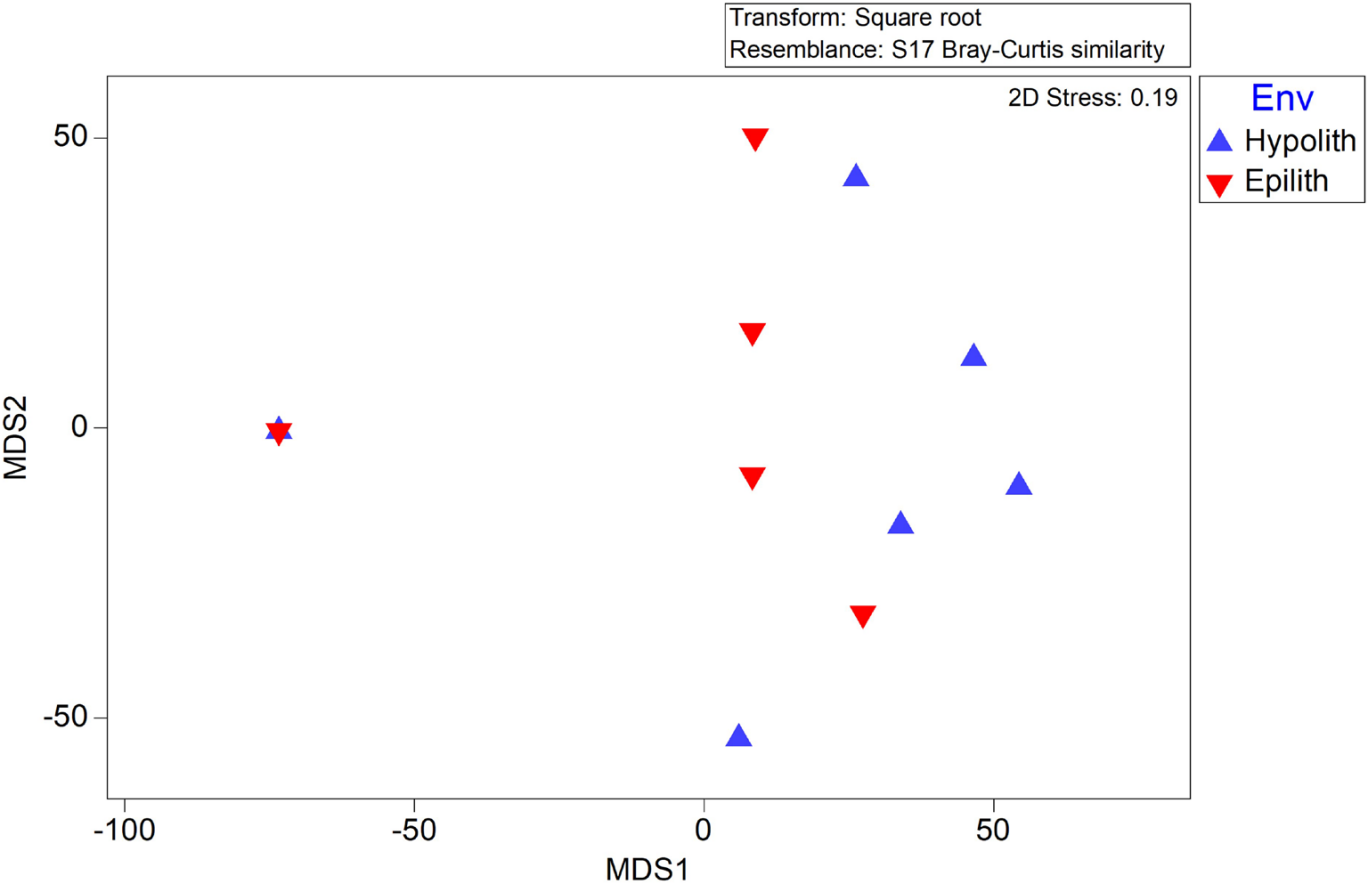
Multi‐Dimensional Scaling plot showing the relative dissimilarity between eukaryotic communities from the hypolithic (blue) and epilithic (red) communities of Arctic rock environments, informed by a Bray–Curtis dissimilarity matrix.

We carried out an identical analysis on the eukaryotic communities associated with the hypolithic and epilithic communities. ANOSIM analysis of eukaryotic communities returned an *R* value of 0.213, indicating no probable difference between the hypolithic and epilithic communities. This is indicated in Figure 5, a multi‐dimensional scaling plot showing the relative distances (similarity) between communities in each environment. Nevertheless, SIMPER analysis did find important indicator species for the hypolithic community (*n* = 7), as shown in Table 3, Table S4, and epilithic communities (*n* = 4), shown in Table S5. SIMPER analysis revealed species which could be used to differentiate between the communities (*n* = 19), as shown in Table 4, Table S6.

**TABLE 3:** Important eukaryotic indicator species associated with the hypolithic community.

Taxon	Description	Av. Abundance	Av. Sim	Sim/SD	Contribution (%)	Cumulative contribution (%)
*Coccomyxa subellipsoidea*	Green alga	17.08	4.43	0.91	18.28	18.28
*Echiniscidea*	Tardigrade	27.64	4.20	0.51	17.34	35.02
Spirotrichea	Ciliate	13.32	3.90	1.12	16.10	51.71
Ulotrichales	Green alga	6.63	1.67	0.86	6.89	58.61
Trebouxiophyceae^1^	Green alga	5.11	0.99	0.79	4.10	62.71
Eimeriidae	Apicomplexa	3.64	0.99	1.18	4.08	66.79
Trebouxiophyceae^2^	Green alga	4.82	0.94	0.75	3.87	70.66

*Note:* Taxon is assigned to the highest identified level. Information includes the taxon and phylum of each species, as well as the average abundance of sequences (Av. Abundance), calculated average similarity (Av. Sim), similarity/standard deviation ratio (Sim/SD), and the SIMPER calculated contribution of each species to overall similarity, which is also given cumulatively. Two taxa within Trebouxiophyceae were identified separately and are marked as 1 and 2, although the exact taxa are unknown.

**TABLE 4:** The top 10 differentiating eukaryotic taxa between hypolithic and epilithic community.

Taxon	Description	Av. Abundance (hypolith)	Av. Abundance (epilith)	Av. Diss	Diss/SD	Contribution (%)	Cumulative contribution (%)
Echiniscidae	Tardigrade	27.64	8.50	9.46	1.00	11.73	11.73
*Coccomyxa subellipsoidea*	Green alga	17.08	22.49	8.97	1.21	11.12	22.85
*Chlorodium saccharophilum*	Green alga	12.19	15.22	5.81	1.19	7.20	30.05
Spirotrichea	Ciliate	13.32	7.14	5.48	0.97	5.80	35.85
Jungermanniales	Liverwort	11.75	8.52	4.93	0.81	5.12	42.98
*Trentepohlia iolithus*	Green alga	4.63	13.85	4.11	1.10	5.10	48.08
Ulotrichales	Green alga	6.63	1.44	2.31	1.18	2.87	50.94
*Aphelenchoides* sp.	Nematode	3.63	0	1.94	0.44	2.40	53.34
*Aphelenchoides*	Nematode	4.85	2.33	1.60	1.16	1.98	55.32
*Neocystis brevis*	Green alga	5.11	0.44	1.54	1.24	1.91	57.24

*Note:* Taxon is assigned to highest identified level. Information includes the taxon and phylum of each species, as well as average abundance of sequences in hypolith and epilith environments (av. Abundance), average dissimilarity (Av. Diss), dissimilarity/standard deviation ratio (Diss/SD), and the SIMPER calculated contribution of each species to overall dissimilarity, which is also given cumulatively.

**FIGURE 6:**
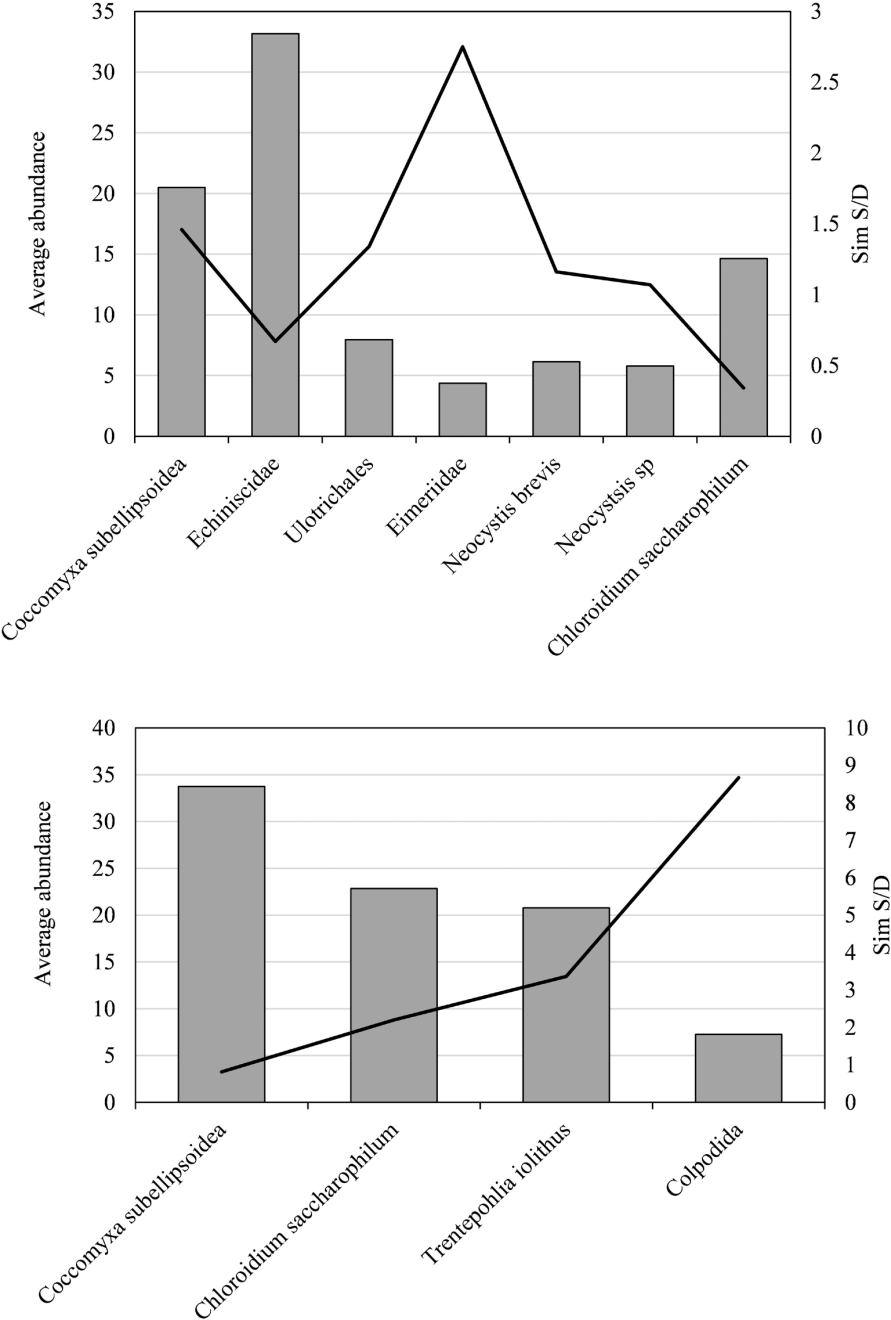
The mean abundance (bars) and statistical importance (line) of each eukaryotic taxa characteristic of hypolithic (upper graph) and epilithic (lower graph) communities from Arctic rock environments. Importance determined by SIMPER analysis.

Figure 6 shows the average abundance and statistical importance of each indicator eukaryotic species in the hypolithic and epilithic community determined by SIMPER analysis. These data are also shown in figures showing the relative importance of each indicator species in characterising each eukaryotic community (Figure S3) and the abundance of each differentiator species in either community (on a logarithmic scale), as well as the importance of that species (Figure S4).

**FIGURE 7:**
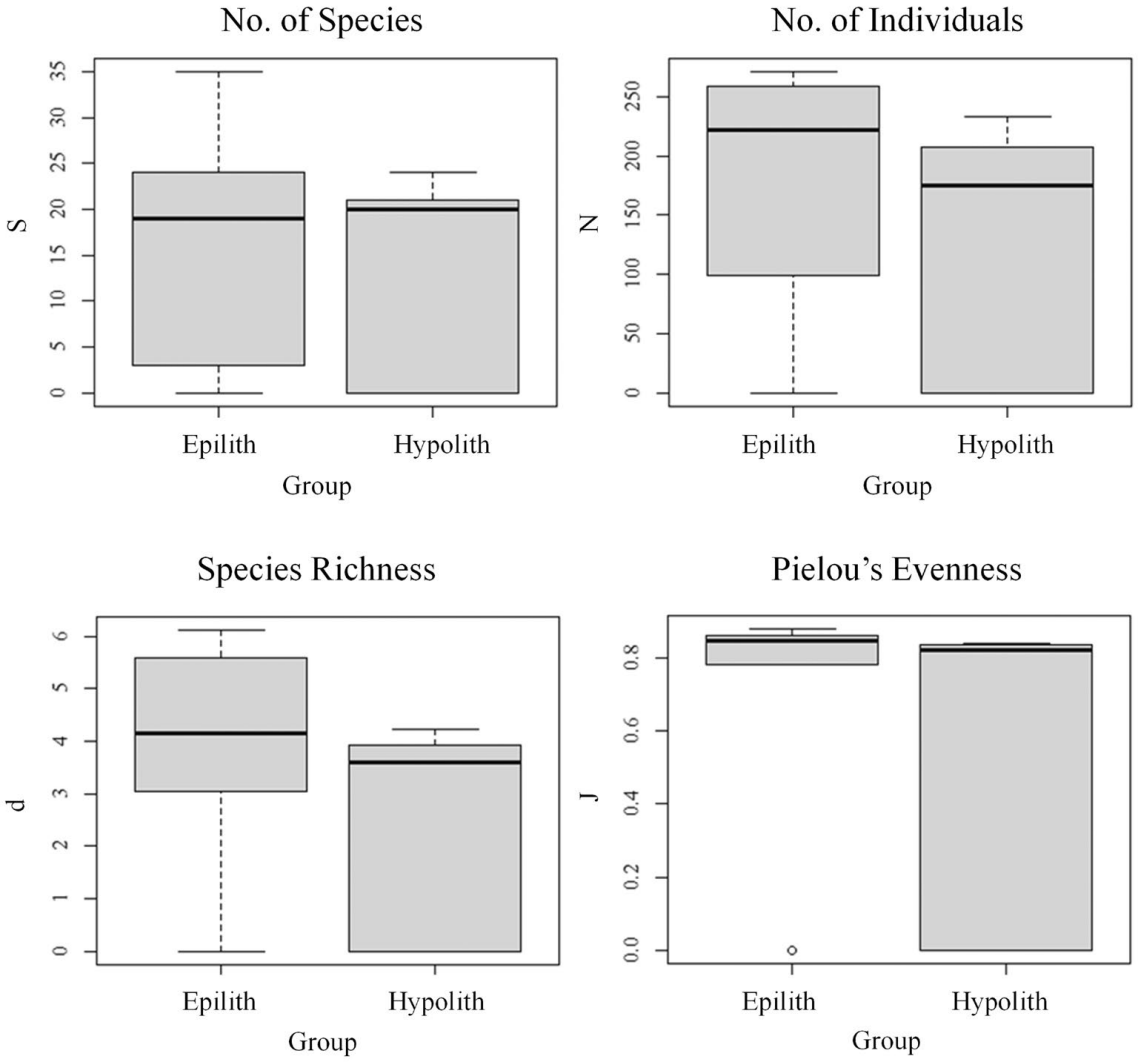
Univariate analysis of eukaryotic communities comparing hypolithic and epilithic communities. Data showing the total number of species (*S*), total number of individuals (*N*), species richness (*d*) and Pielou's species evenness (*J*).

As with prokaryotic communities, the eukaryotic data can be better understood by examining the univariate statistics shown in Figure 7. The mean *S* scores were: hypolithic, 21.5 ± 11.59; eplithic, 14.17 ± 10.12. The mean *N* scores were: hypolithic, 179.07 ± 99.50; epilithic, 131.65 ± 95.57. The mean *d* scores were: hypolithic, 3.84 ± 2.00; epilithic, 2.56 ± 1.83. The mean *J* scores were: hypolithic: 0.702 ± 0.316; epilithic: 0.552 ± 0.391. Pairwise Welch two‐sample *t*‐tests revealed no significant differences between the communities in any measure (*p* > 0.05).

Sequences affiliated with fungi were observed in both hypolithic and epilithic communities, but in all cases, they constituted < 3% of the sequences. In both habitats, sequences affiliated with Ascomycota dominated the community, with sequences matching *Ramularia*, *Verrucaria*, and *Isaria* spp. In the epilithic habitats, sequences affiliated with lichenous fungi were observed, including sequences that closely affiliated with *Abrothallus* and *Lecidea*. In both habitats, sequences affiliated with Chytridiomycetes were observed, including sequences most closely matching Spizellomycetales and Rhizophydiales.

## 4 Discussion

Rocks across extensive regions of polar deserts host communities on their surfaces that exhibit sharp visual demarcation between green bands on the underside of the rocks and dark black biofilms inhabiting the top surface (Cockell and Stokes 2004, 2006). The underside of these usually opaque rocks can be colonized by photosynthetic microorganisms because periglacial processes move the rocks, opening up space on the rock underside into which light can penetrate. On Devon and Cornwallis Islands, Canadian High Arctic, Cockell and Stokes (2006) found that 95% and 94% of rocks in patterned ground (polygons) were colonized by hypoliths on the islands, respectively. For all Arctic polygonal rock‐sorted ground, 68% of rocks were colonized within the polygons. The mean widths of the hypolithic (green) subsurface growth bands in patterned ground were 3.1 ± 1.9 cm on Devon Island and 3.0 ± 1.6 cm on Cornwallis Island.

In this work, we wanted to explore the hypothesis that the surface epilithic community is different from the hypolithic community on the underside of the rocks. The hypolithic habitat is known to host relatively clement physical conditions compared to the surface in extreme deserts (Chan et al. 2012), and so we might expect those differences to drive community differences.

Using 16S rDNA analysis, we were able to show by ANOSIM analysis of bacterial communities that there was a highly significant difference between the hypolithic bacterial community and the surface epilithic community. SIMPER analysis further revealed that *Chroococcidiopsis* was the most abundant taxon in the hypolithic community, consistent with this being a phototroph‐dominated community. The hypolithic habitat was found to support the growth of *Gloeobacter violaceus*, previously reported as a primitive rock‐dwelling cyanobacterium (Mareš et al. 2013). The photosynthetic organisms we found in the hypolithic habitat and on the surface of the rocks were similar to those observed in other hot and cold desert environments including Chroococcales, Nostocales, and Pleurocapsales (e.g., Chan et al. 2012; Makhalanyane, Valverde, Birkeland, et al. 2013; Makhalanyane, Valverde, Lacap, et al. 2013). Nostocales have previously been shown to be a dominant taxon in Antarctic hypoliths (Pointing et al. 2009; Khan et al. 2011; Wood et al. 2008; Lebre et al. 2021).

Heterotrophs were abundant. Alphaproteobacteria was an abundant class, which dominated the Pseudomonadota and had sequences affiliated with Sphingomonadales (e.g., *Sphingomonas* and *Sphingosinicella* spp.) as the second most abundant taxon in the hypolithic community. *Sphingomonas* spp. have previously been shown to be associated with polar lichens (Lee et al. 2014). Other alphaproteobacteria were found to be prominent, including Rhodospirillales (e.g., *Acidisphaera* and *Roseomonas* spp.) and Caulobacterales. Also prominent were Actinobacteria (e.g., *Pseudonocardia* and *Frankia* spp.), which have previously been reported from cold and dry glaciated environments (Gonzalez‐Toril et al. 2008). Acidobacteria (*Acidobacterium* spp) were prominent and have previously been associated with carbon cycling in arctic tundra (Männistö et al. 2013; Choe et al. 2018) and, with the organisms discussed above, may play an important role in carbon cycling in the widespread arctic hypolithic biome. A plausible source of organics for these organisms is the hypolithic phototrophic community on the same rock or organics transported in meltwater through the patterned ground. Our data were qualitatively similar to those obtained by Lebre et al. (2021) who found that in Antarctic hypoliths from the McMurdo Dry Valleys, bacterial heterotrophic taxa were dominated by Proteobacteria (Pseudomonadota) and Actinobacteria within a community overall dominated by Cyanobacteria. Similar results showing a dominance of Actinobacteria and Proteobacteria in the heterotrophic population were found in diverse lithologies from Svalbard, Norwegian High Arctic, by Choe et al. (2018), who similarly to us, found Bacteroidetes to be a low abundance phylum.

The differences in the heterotrophic community between the hypolithic and epilithic communities were primarily driven by differences in abundance rather than differences in community structure. One prominent taxon that was found exclusively on the surface of the rocks and not in the hypolithic habitat was *Hymenobacter*, members of which from Antarctic regolith have been shown to have high UV radiation resistance (Sedláček et al. 2019), attributes which might make species of this genus suitable colonists for the surface of the arctic rocks.

Our data support the hypothesis that, at least with respect to the bacteria, the physical conditions in the hypolithic and epilithic environments select for two distinct communities. These differences in physical conditions may include the more abundant availability of water in the hypolithic environment. During July and August, this was often observed as snowmelt which percolated along the undersides and edges of patterned ground to the hypoliths while the surface of the rocks, except during rainfall events, generally remained windswept and dry. Nevertheless, some species are common to both communities such as *Chroococcidiopsis* spp., a ubiquitous denizen of extreme hot and cold deserts around the world (Bahl et al. 2011; Grilli Caiola et al. 1993; Lacap et al. 2011) and quartzitic hypolithic habitats (Lacap‐Bugler et al. 2017). *Chroococcidiopsis* sp. is a photosynthetic extremophile (Baqué et al. 2013), known to survive in both low and high temperature conditions, as well as having high tolerance to ionizing radiation (Billi et al. 2000), hypersaline environments (Cumbers and Rothschild 2014) and desiccating conditions (Billi et al. 2000). *Chroococcidiopsis* sp. has been previously recorded on translucent rocks with highly variable light penetration (Smith et al. 2000). The species common to both communities may be cosmopolitan species capable of growing under a range of extremes and therefore not differentiated in the two micro‐environments on the rock.

In terms of the sharp demarcation in pigmentation observed between the hypoliths and epilithic communities, our results leave open the possibility that either the same organisms in both communities may be physiologically responding differently (since there is a taxonomic overlap between the inhabitants in the two communities) or that different organisms are involved in the black pigmentation on the surface. The cyanobacterial UV‐screening pigment scytonemin (Sinha and Häder 2008; Soule et al. 2009) has been reported to have a specific threshold of induction of 33 μmole photons m^−2^ s^−1^ (Garcia‐Pichel and Castenholz 1991). Such threshold effects may explain the switch from the green pigmented hypolithic community and the black surface community over small (millimetre) scales, since one could hypothesize that certain taxa, such as *Chroococcidiopsis*, present on both the underside and top surface, are triggered to produce pigments at a critical light threshold on the rock. By microscopy, the black pigmented organisms on the surface are observed to be primarily coccoid (data not shown) consistent with taxa such as *Chroococcidiopsis* and *Gloeobacter* spp. found on both surfaces. Other taxa that are known to produce UV‐screening pigments, such as *Nostoc* spp. (Ferreira and Garcia‐Pichel 2016) are present on both surfaces. Within our data, there is no obvious presence of dominant taxa that are known to produce dark UV‐screening pigments such as scytonemin or gloeocapsin on the surface of the rock that are not present in the hypolithic community. However, we cannot rule out strains or species on the surface that produce UV‐screening pigments that are not present on the underside.

While a holistic view of the communities can be achieved by applying multivariate methods, several univariate analyses can provide deeper insights into the community. The higher *S* value in the hypolithic habitat, indicating the number of species, could reflect more active phototrophy (and concomitantly potentially more active C‐ and N‐fixation) compared to the surface community, which is generally drier and where organisms may direct more energy into pigment production, although this hypothesis would have to be tested with direct activity measurements on both sides of the rock.

More specifically, when we take into account the *N* value (the total number of organisms of all species in each environment), which is roughly the same in both communities, it could imply that the exposed surface of the rock is home to a few, highly specialised genera in high abundance, which are then marginalised in the hypolithic community by species which are less adapted to extreme conditions, such as radiation or desiccation tolerance. We can therefore draw two potential conclusions from these indices: the bottom surface of arctic rocks acts as a refuge against extreme environments for a greater variety of species, and certain species which are well adapted to the extreme conditions are able to grow in relatively high abundance even on the exposed surface. These values are combined in the Margalef species richness index (*d*), which supports these conclusions.

Although the total number of species, *N*, was similar in the hypolithic and surface communities, Pielou's Evenness index (*J*) indicates how well distributed the total population is across different species within a community. If all species in a given community have the same population size, then the value of *J* would be 1.

The high *J* values in both hypolithic and epilithic communities (both > 0.8) are indicative of relatively stable ecosystems in both cases; in an unstable ecosystem, we would perhaps see a single opportunistic species dominate. This is further demonstrated by the Margalef species richness index (*d*), which takes into account both the number of individuals and the number of species (*S*). The *d* value is relatively high in both communities, but the value is significantly higher in the hypolithic community. This may indicate a greater species evenness in the hypolithic community and perhaps greater ecosystem stability.

We observed variation between rocks, despite the presence of dominant ‘core’ taxa that define the general microbial characteristics of the Arctic hypolithic habitat. These differences likely reflect micro‐environmental differences between rocks (but broadly shared macro‐environmental conditions), and/or they could reflect stochastic processes involved in the initial colonization of rocks (founder effects), such as dispersal limitations, and the interactions between species that colonize the rocks, as has been observed to be the case in Antarctic hypoliths (Lebre et al. 2021).

The eukaryotic communities in the hypolithic and epilithic habitats, similarly analysed, are not statistically different from one another. Among eukaryotic taxa, green algae are an abundant component of the phototrophic community, dominated by Coccomyxaceae and Trebouxiophyceae. These taxa have been shown to be components of lichens (Chen et al. 2024) and free‐living organisms (Kania et al. 2023) in polar microbial communities. Trebouxiophyceae are found as rock‐dwelling eukaryotic components of Antarctic endoliths (Martins et al. 2020; Clarke et al. 2024) suggesting their adaptations to polar conditions.

We observed fungal sequences within the epilithic and hypolithic communities. Although they were always < 3% of the sequences, they have a potentially important role to play. Some sequences affiliated with lichenous fungi, such as *Abrothallus* and *Lecidea*, and given the observation of sequences of Trebouxiophycaea, which can be an algal lichen symbiont, this may suggest that in the epilithic community some algae and fungi form a lichenous association (although macroscopically they do not form contiguous lichenous growth). Ascomycota have previously been shown to inhabit hypolithic habitats in the Antarctic (Gokul et al. 2013) and similarly to Gokul and co‐workers, we observed sequences affiliated to *Verrucaria* spp. In both habitats, sequences affiliated with Chytridiomycetes were observed. These taxa have been shown to be involved in biomass degradation, including in high‐altitude soils (Freeman et al. 2009) and in the polar rocky environment they may play an important role in the heterotrophic cycling of organic matter. Our genetic analysis was limited to 18S rDNA analysis, but our results show that more research on the role of fungi in carbon and biomass cycling in high arctic deserts is warranted, since in a warmer polar future, they may play important roles in carbon cycling.

Intriguingly, the multivariate SIMPER analysis indicates that the hypolithic community was defined by the presence of Animalia, including nematodes and Echiniscidae (tardigrades), the latter being known for their endurance of extreme environments (Carrero et al. 2019). Despite attempts to do so, we were unable to culture tardigrades in the laboratory from the samples (data not shown), but they plausibly feed on the sometimes‐dense microbial biofilms that form on the underside of the rocks in the presence of the snowmelt collecting around the edges of the rocks in the patterned ground. These data show that the micro‐scale spatial differentiation between the hypolithic and epilithic communities can generate sufficient environmental conditions to support more diverse and complex life in the hypolithic environment. In this specific case, millimetre‐scale differences mean the difference between supporting complex multicellular animal life and not.

Although there is no clear statistical difference between the two eukaryotic communities, there may still be a biological difference—the number of species was generally higher in the hypolithic community compared to the epilithic community, perhaps reflecting the presence of a more diverse range of taxa that includes the Animalia. This is supported by the number of individuals, which is relatively higher in the hypolithic community compared to the epilithic community. The Margalef species richness indices reflect this difference in score; a higher mean, and generally higher values overall, for species richness was observed in the hypolithic community. The Pielou's indices have relatively similar means; however, like the prokaryotic communities, the values for the communities varied between samples, suggesting a core set of eukaryotic taxa, but the potential for different communities to assemble on different rocks caused by local environmental differences, founder effects, etc.

Light levels are usually attenuated in the hypolithic habitat compared to the surface, for example, under quarzitic substrates (Smith et al. 2014; Tracy et al. 2010; Warren‐Rhodes et al. 2013). Our results show that although the surface of the arctic rocks must receive more solar energy than the underside, the diversity is higher on the underside. Our results are consistent with those reported by Gwizdala et al. (2021). They suggest that factors other than light exposure, such as water activity, ultimately control the photosynthetic capacity of hypolithic communities. Factors such as snow melt, and thus greater water availability and less UV radiation stress on the underside may account for this higher microbial diversity (and the presence of animal life) in the hypolithic community. Cockell and Stokes found that over a year (from August 2001 to August 2002) a short period of time existed when sunlight was available and mean air temperature was above zero (18 June to 11 July 2002), suggesting that transient liquid water availability on the underside of the rock may allow for hypolithic growth at a time when the surface of the rock may be windblown and dry (and either frozen or in darkness for the remainder of the year). Furthermore, they recorded an instance of the hypolithic environment, because of the thermal inertia of the ground/rock, maintaining temperatures above zero while the air temperature (and thus potentially the temperature on the surface of the rock) fell below zero. Thus, the hypolithic habitat may provide some buffer against freeze–thaw events during the summer. More generally, these data show that energy is not the only determinant of microbial zonation in extreme rocky environments, and that other physical factors play a role in such zonation, allowing for greater organismal diversity in extreme environments which are otherwise more energy starved than less diverse habitats.

**FIGURE 8:**
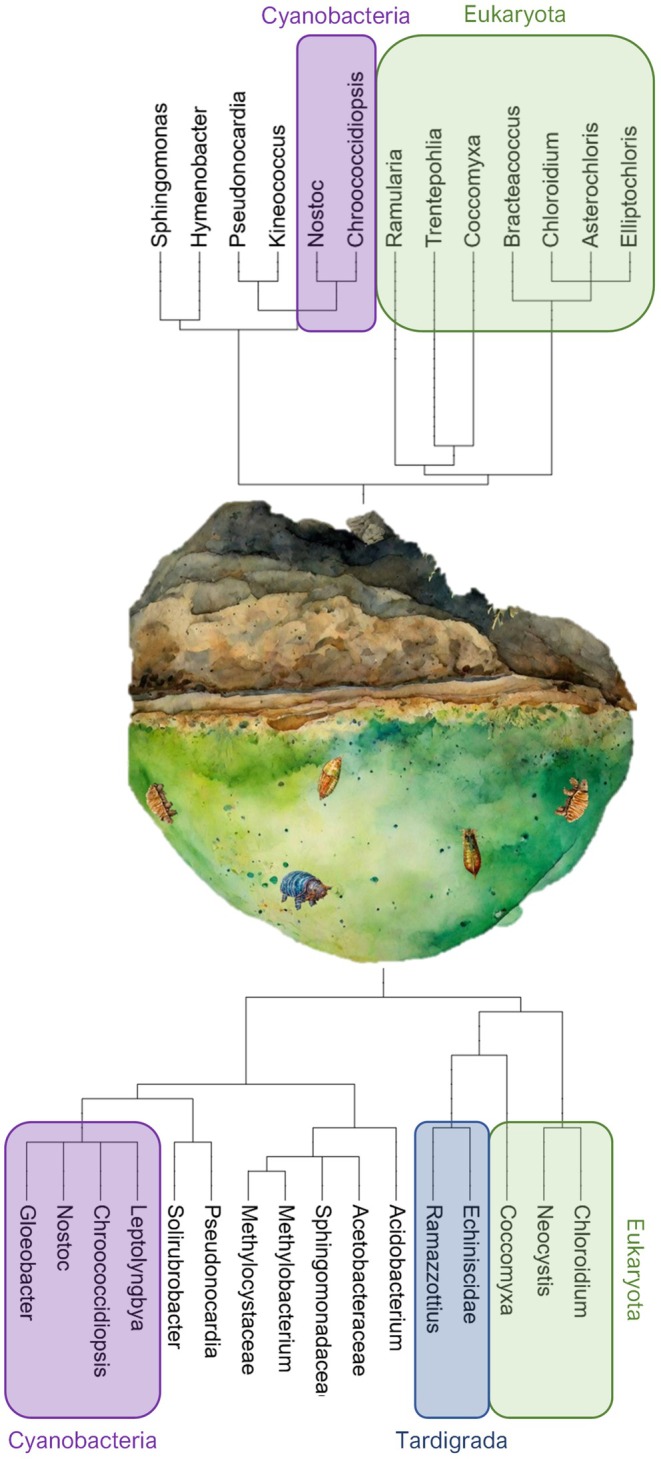
Phylogenetic trees of the characteristic taxa from hypolithic and epilithic communities, as determined by SIMPER analysis and shown alongside a pictorial illustration of the two communities (with animal DNA in the hypolithic community depicted using schematics of these organisms). Taken from SIMPER analysis of combined bacterial and eukaryotic datasets. Colours indicates phylogenetic groupings; Purple = cyanobacteria, Green = eukaryotes, Blue = animalia (Tardigrada) separated from other eukaryotes to emphasize their presence. All others are bacteria.

In Figure 8, we summarize our findings in pictorial form to illustrate the differences and similarities between the two communities that inhabit Arctic rocks.

## 5 Conclusions

The sharp visual demarcation between microbial communities inhabiting the surfaces and subsurfaces of many rocks in polar deserts raises the question of whether they represent two distinct communities. Our results show that these communities are distinct. However, some organisms are common to both, and they likely are more cosmopolitan species. These common species leave open the possibility that pigmentation differences between hypolithic and epilithic communities could be caused by physiological responses, for example to higher ultraviolet radiation fluxes on the surface of the rock, by the same species represented in both microhabitats. This study leaves open several other questions. Many of the species remain “Unknown” or “Unclassified”; more detailed sequence analysis could elucidate these novel species and provide further insight on the community function and composition of these polar ecosystems.

## Conflicts of Interest

The authors declare no conflicts of interest.

## Supporting information


Data S1.

## Data Availability

The data that support the findings of this study are available from the corresponding author upon reasonable request.
