# Supplementary material for: Two Worlds on a Stone: Arctic Desert Hypoliths and Epiliths Show Spatial Niche Differentiation
Source: Geobiology. 2025 Jun 26;23(4):e70025. doi: 10.1111/gbi.70025 (PMC12199873; doi:10.1111/gbi.70025)
Supplement: Supplementary file 1 — Data S1. [file GBI-23-e70025-s001.docx]

# Two worlds on a stone: Arctic desert hypoliths and epiliths show spatial niche differentiation

SUPPLEMENTAL FIGURES.


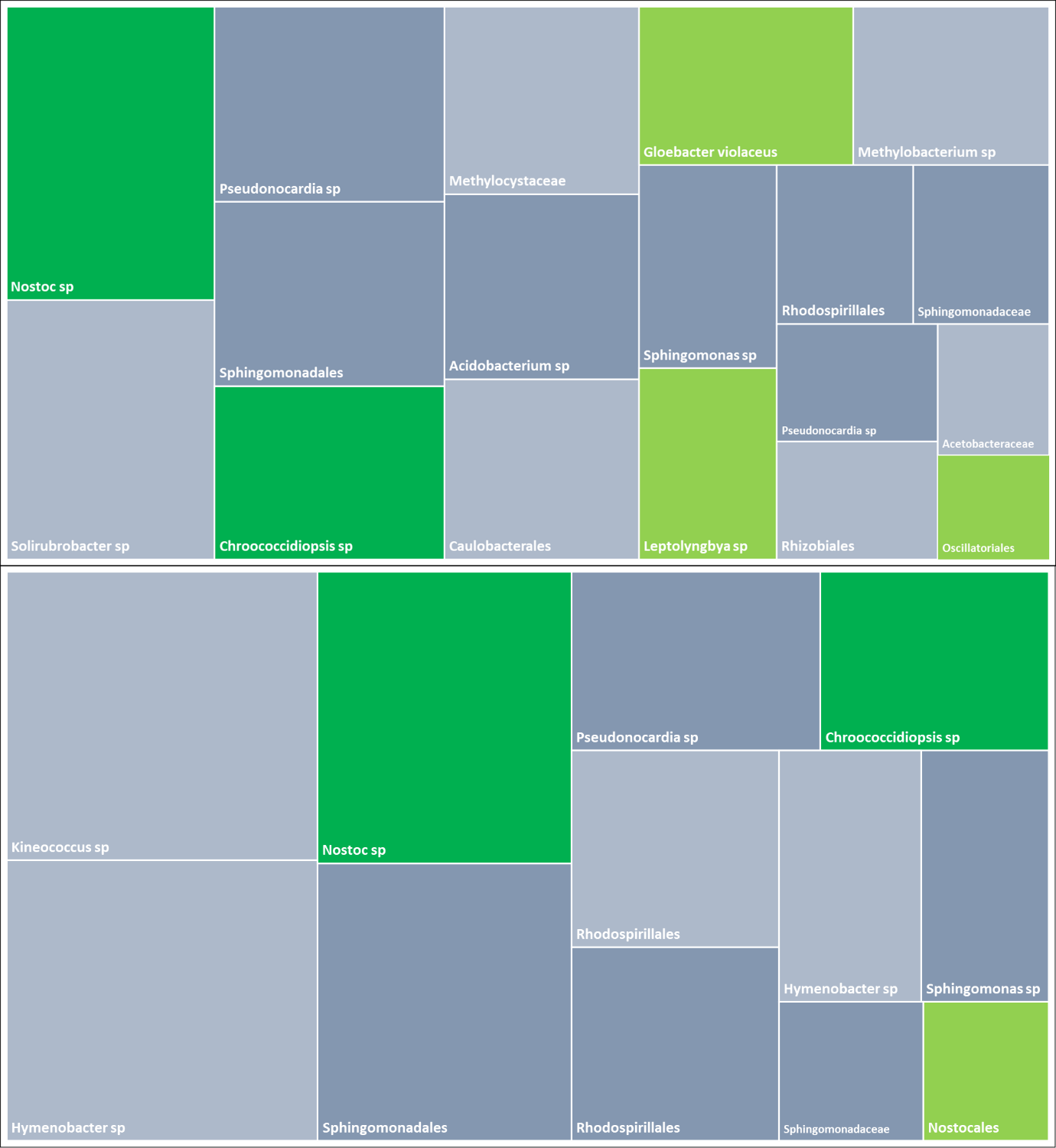
 Supplementary Figure 1. Relative importance plot. The relative importance of taxa in characterising hypolithic (upper diagram) and epilithic (lower diagram) communities. Taxa highlighted in green represent Cyanobacteria. Taxa which appear darker are characteristic of both environments.


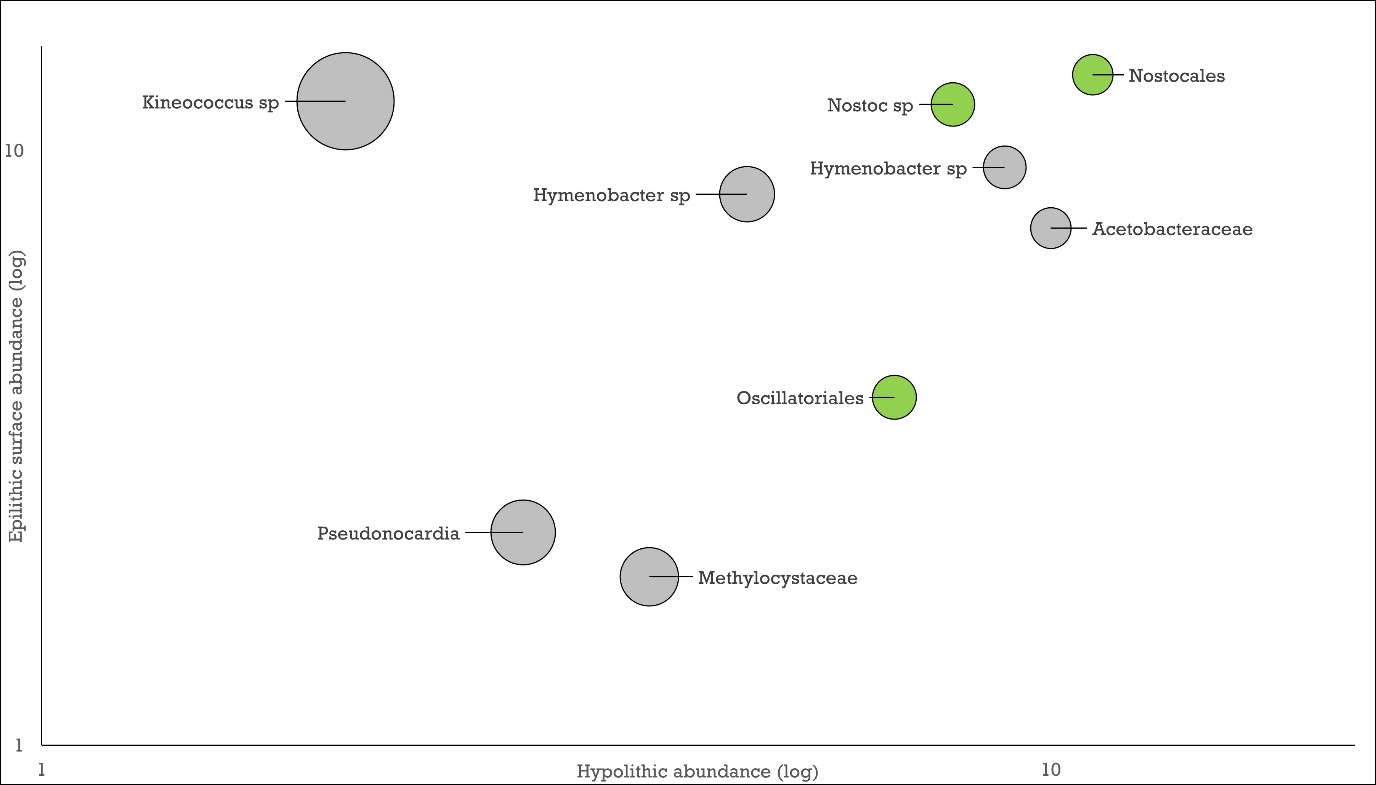
 Supplementary Figure 2. The relative abundance of prokaryotic taxa in hypolithic and epilithic communities, which are significant differentiators between the environments. Bubble size represents the relative importance of that taxa as a differentiator, as revealed by SIMPER analysis. Taxa highlighted in green represent Cyanobacteria.

*
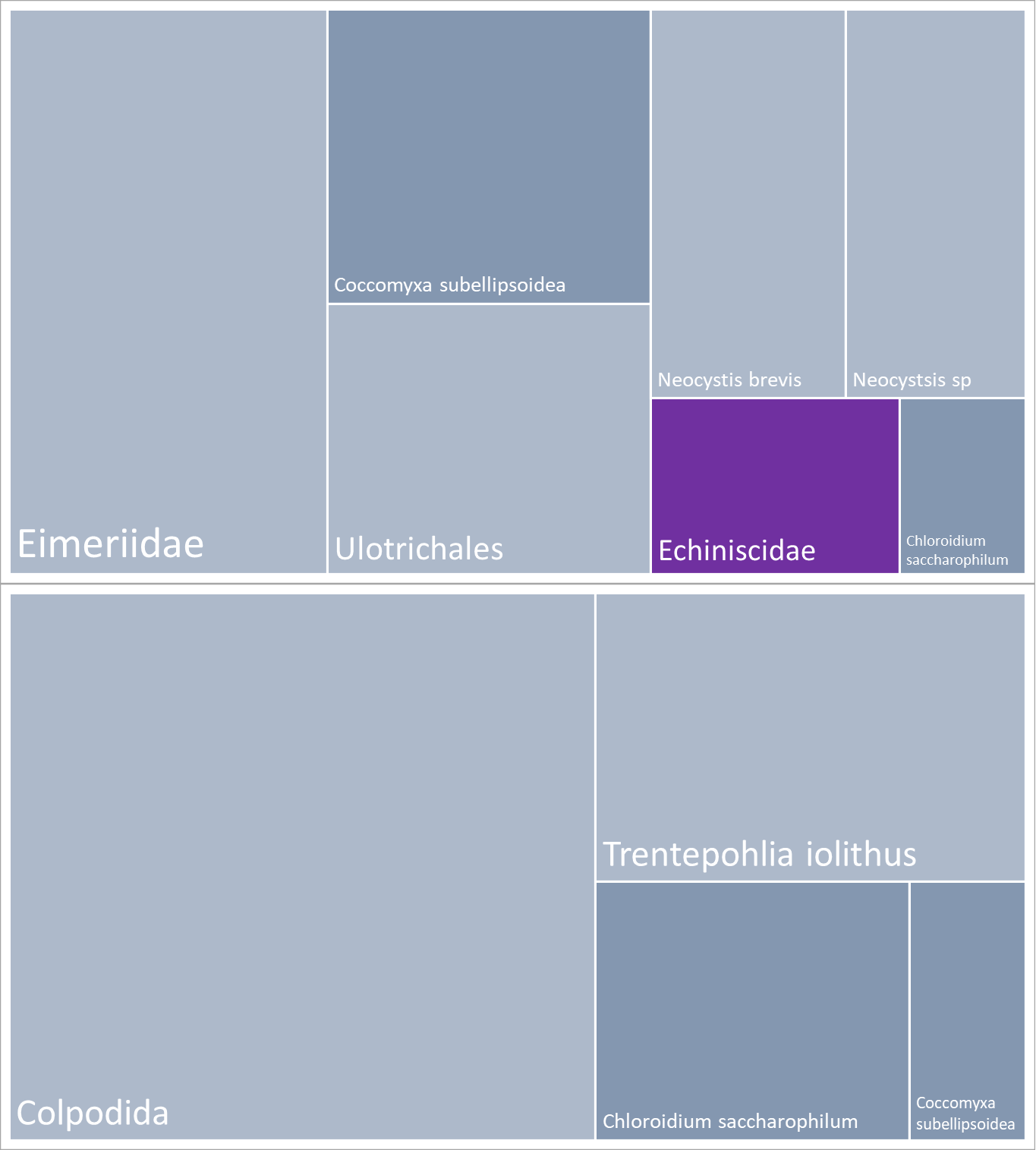
*

Supplementary Figure 3. The relative importance of eukaryotic taxa in characterising hypolithic (upper diagram) and epilithic (lower diagram) communities. Taxa highlighted in purple represent Animalia. Taxa which appear darker are characteristic of both environments.

**
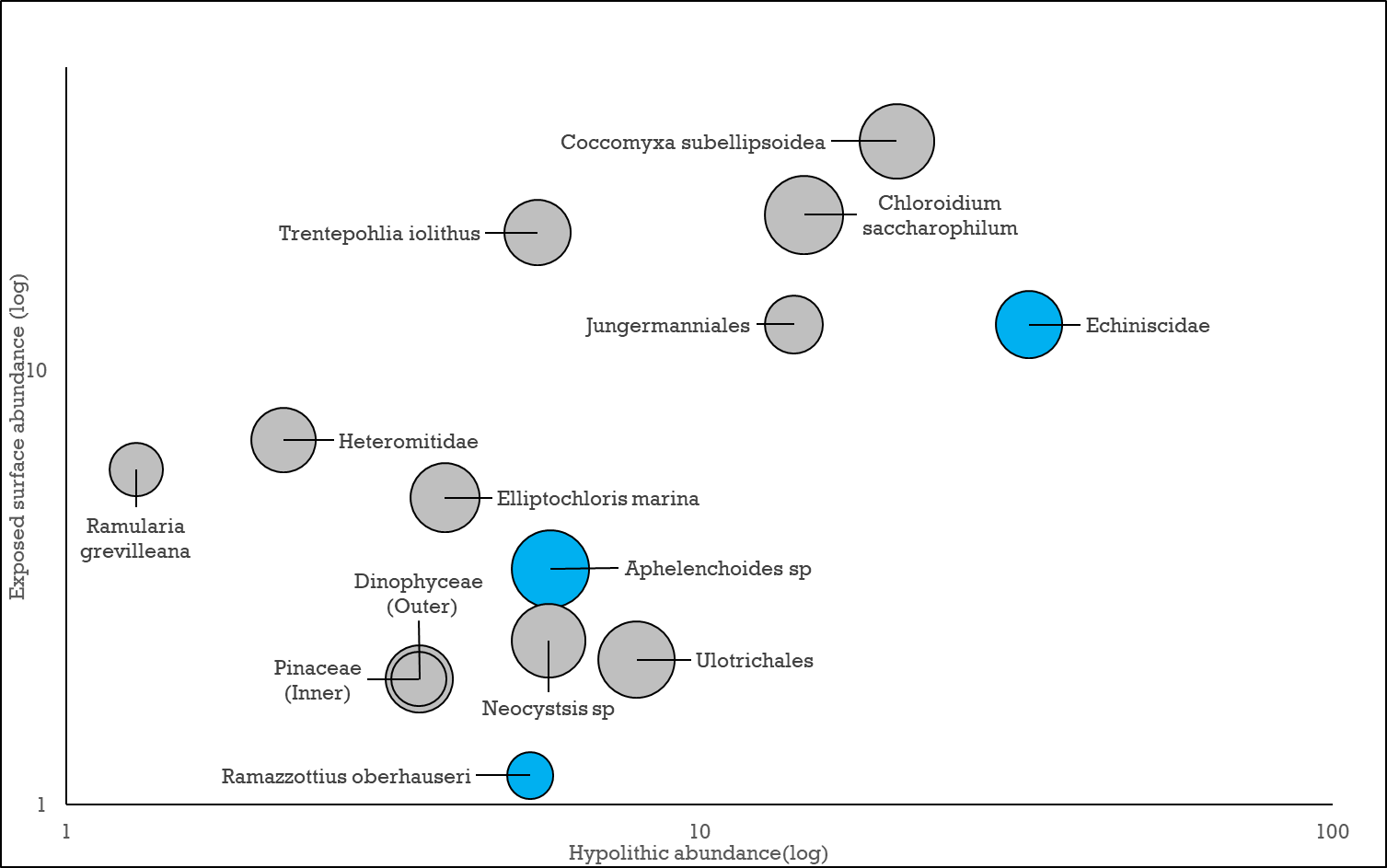
**

Supplementary Figure 4. The relative abundance of eukaryotic taxa in hypolithic and epilithic communities, which are significant differentiators between the environments. Bubble size represents the relative importance of that taxa as a differentiator, as revealed by SIMPER analysis. Taxa highlighted in blue represent Animalia.

SUPPLEMENTAL TABLES.

| Supplementary Table 1.- Indicator species of Hypolith communities as determined by SIMPER analysis. Average Similarity=66.27 | | | | | |
| --- | --- | --- | --- | --- | --- |
| Species | Av.Abund | Av.Sim | Sim/SD | Contrib% | Cum.% |
| Bacteria ; Cyanobacteria ; Unclassified ; Pleurocapsales ; Unclassified ; Chroococcidiopsis ; Chroococcidiopsis sp | 34.69 | 8.14 | 6.1 | 12.29 | 12.29 |
| Bacteria ; Proteobacteria ; Alphaproteobacteria ; Sphingomonadales ; Unknown ; Unknown ; Unknown | 17.26 | 3.79 | 6.6 | 5.72 | 18.01 |
| Bacteria ; Proteobacteria ; Alphaproteobacteria ; Sphingomonadales ; Sphingomonadaceae ; Sphingomonas ; Sphingomonas sp | 16.73 | 3.56 | 4.37 | 5.37 | 23.38 |
| Bacteria ; Actinobacteria ; Actinobacteria ; Pseudonocardiales ; Pseudonocardiaceae ; Pseudonocardia ; Pseudonocardia sp | 16.25 | 3.51 | 7.08 | 5.3 | 28.68 |
| Bacteria ; Proteobacteria ; Alphaproteobacteria ; Sphingomonadales ; Sphingomonadaceae ; Unknown ; Unknown | 16.52 | 3.31 | 3.34 | 4.99 | 33.67 |
| Bacteria ; Actinobacteria ; Actinobacteria ; Pseudonocardiales ; Pseudonocardiaceae ; Pseudonocardia ; Unknown | 14.56 | 3.2 | 2.98 | 4.83 | 38.5 |
| Bacteria ; Cyanobacteria ; Gloeobacteria ; Gloeobacterales ; Unclassified ; Gloeobacter ; Gloeobacter violaceus | 14.21 | 2.88 | 5.22 | 4.35 | 42.86 |
| Bacteria ; Proteobacteria ; Alphaproteobacteria ; Rhodospirillales ; Unclassified ; Unclassified ; Unclassified | 12.85 | 2.43 | 3.22 | 3.67 | 46.53 |
| Bacteria ; Acidobacteria ; Acidobacteriia ; Acidobacteriales ; Acidobacteriaceae ; Acidobacterium ; Acidobacterium sp | 9.65 | 2.14 | 5.72 | 3.23 | 49.76 |
| Bacteria ; Proteobacteria ; Alphaproteobacteria ; Rhodospirillales ; Acetobacteraceae ; Unknown ; Unknown | 11.27 | 1.99 | 2.26 | 3 | 52.76 |
| Bacteria ; Cyanobacteria ; Unclassified ; Oscillatoriales ; Unclassified ; Unknown ; Unknown | 10.57 | 1.84 | 1.78 | 2.78 | 55.54 |
| Bacteria ; Proteobacteria ; Alphaproteobacteria ; Rhizobiales ; Methylocystaceae ; Unknown ; Unknown | 7.32 | 1.68 | 5.77 | 2.53 | 58.07 |
| Bacteria ; Proteobacteria ; Alphaproteobacteria ; Caulobacterales ; Unknown ; Unknown ; Unknown | 6.26 | 1.28 | 5.36 | 1.93 | 60 |
| Bacteria ; Cyanobacteria ; Unclassified ; Nostocales ; Nostocaceae ; Nostoc ; Nostoc sp | 6.23 | 1.23 | 9.36 | 1.86 | 61.86 |
| Bacteria ; Actinobacteria ; Thermoleophilia ; Solirubrobacterales ; Solirubrobacteraceae ; Solirubrobacter ; Solirubrobacter sp | 5.36 | 1.11 | 8.14 | 1.68 | 63.54 |
| Bacteria ; Proteobacteria ; Alphaproteobacteria ; Rhizobiales ; Methylobacteriaceae ; Methylobacterium ; Unknown | 5.42 | 1.1 | 4.63 | 1.66 | 65.2 |
| Bacteria ; Proteobacteria ; Alphaproteobacteria ; Rhizobiales ; Unknown ; Unknown ; Unknown | 5.29 | 1 | 2.93 | 1.51 | 66.71 |
| Bacteria ; Cyanobacteria ; Unclassified ; Oscillatoriales ; Unclassified ; Leptolyngbya ; Unknown | 5.26 | 0.98 | 3.99 | 1.48 | 68.19 |
| Bacteria ; Acidobacteria ; Acidobacteriia ; Acidobacteriales ; Unclassified ; Unclassified ; Unclassified | 4.69 | 0.95 | 5.9 | 1.44 | 69.63 |
| Bacteria ; Proteobacteria ; Alphaproteobacteria ; Rhodospirillales ; Unknown ; Unknown ; Unknown | 5.1 | 0.93 | 3.19 | 1.4 | 71.03 |

| Supplementary Table 2.- Indicator species of Epilith communities as determined by SIMPER analysis. Average Similarity=67.09 | | | | | |
| --- | --- | --- | --- | --- | --- |
| Species | Av.Abund | Av.Sim | Sim/SD | Contrib% | Cum.% |
| Bacteria ; Cyanobacteria ; Unclassified ; Pleurocapsales ; Unclassified ; Chroococcidiopsis ; Chroococcidiopsis sp | 27.38 | 6.57 | 4.02 | 9.79 | 9.79 |
| Bacteria ; Proteobacteria ; Alphaproteobacteria ; Rhodospirillales ; Unclassified ; Unclassified ; Unclassified | 32.03 | 6 | 3.69 | 8.95 | 18.74 |
| Bacteria ; Proteobacteria ; Alphaproteobacteria ; Sphingomonadales ; Sphingomonadaceae ; Sphingomonas ; Sphingomonas sp | 19.75 | 4.58 | 3.21 | 6.83 | 25.57 |
| Bacteria ; Actinobacteria ; Actinobacteria ; Pseudonocardiales ; Pseudonocardiaceae ; Pseudonocardia ; Pseudonocardia sp | 20.03 | 4.54 | 4.46 | 6.77 | 32.34 |
| Bacteria ; Proteobacteria ; Alphaproteobacteria ; Sphingomonadales ; Unknown ; Unknown ; Unknown | 16.6 | 4.01 | 7.25 | 5.97 | 38.31 |
| Bacteria ; Proteobacteria ; Alphaproteobacteria ; Sphingomonadales ; Sphingomonadaceae ; Unknown ; Unknown | 16.82 | 3.68 | 1.96 | 5.48 | 43.79 |
| Bacteria ; Actinobacteria ; Actinobacteria ; Kineosporiales ; Kineosporiaceae ; Kineococcus ; Unknown | 12.1 | 3.11 | 9.58 | 4.64 | 48.43 |
| Bacteria ; Cyanobacteria ; Unclassified ; Nostocales ; Nostocaceae ; Nostoc ; Nostoc sp | 11.96 | 2.97 | 7.4 | 4.43 | 52.87 |
| Bacteria ; Cyanobacteria ; Unclassified ; Nostocales ; Unknown ; Unknown ; Unknown | 13.41 | 2.97 | 1.68 | 4.43 | 57.29 |
| Bacteria ; Proteobacteria ; Alphaproteobacteria ; Rhodospirillales ; Unknown ; Unknown ; Unknown | 11.52 | 2.57 | 3.81 | 3.83 | 61.12 |
| Bacteria ; Bacteroidetes ; Cytophagia ; Cytophagales ; Cytophagaceae ; Hymenobacter ; Unknown | 9.37 | 1.84 | 3.26 | 2.74 | 63.86 |
| Bacteria ; Bacteroidetes ; Cytophagia ; Cytophagales ; Cytophagaceae ; Hymenobacter ; Hymenobacter sp | 8.45 | 1.84 | 7.96 | 2.74 | 66.6 |
| Bacteria ; Acidobacteria ; Acidobacteriia ; Acidobacteriales ; Acidobacteriaceae ; Acidobacterium ; Acidobacterium sp | 8.05 | 1.71 | 3.42 | 2.55 | 69.15 |
| Bacteria ; Cyanobacteria ; Unclassified ; Nostocales ; Nostocaceae ; Nostoc ; Unknown | 8.97 | 1.71 | 1.2 | 2.55 | 71.7 |

| Supplementary Table 3. Differentiator species between Epilith and Hypolith communities as determined by SIMPER analysis. Average Dissimilarity=44.78 | | | | | |  |
| --- | --- | --- | --- | --- | --- | --- |
| Species | Av. Hypolith Abund | Av. Epilith Abund | Av.Diss | Diss/SD | Contrib% | Cum.% |
| Bacteria ; Proteobacteria ; Alphaproteobacteria ; Rhodospirillales ; Unclassified ; Unclassified ; Unclassified | 12.85 | 32.03 | 2.61 | 1.2 | 5.82 | 5.82 |
| Bacteria ; Actinobacteria ; Actinobacteria ; Pseudonocardiales ; Pseudonocardiaceae ; Pseudonocardia ; Unknown | 14.56 | 2.28 | 1.67 | 2.99 | 3.74 | 9.56 |
| Bacteria ; Cyanobacteria ; Unclassified ; Nostocales ; Unknown ; Unknown ; Unknown | 2.96 | 13.41 | 1.5 | 1.83 | 3.36 | 12.92 |
| Bacteria ; Cyanobacteria ; Unclassified ; Pleurocapsales ; Unclassified ; Chroococcidiopsis ; Chroococcidiopsis sp | 34.69 | 27.38 | 1.28 | 1.5 | 2.86 | 15.77 |
| Bacteria ; Actinobacteria ; Actinobacteria ; Kineosporiales ; Kineosporiaceae ; Kineococcus ; Unknown | 3.92 | 12.1 | 1.12 | 4.45 | 2.5 | 18.27 |
| Bacteria ; Cyanobacteria ; Gloeobacteria ; Gloeobacterales ; Unclassified ; Gloeobacter ; Gloeobacter violaceus | 14.21 | 5.94 | 1.1 | 1.8 | 2.47 | 20.74 |
| Bacteria ; Bacteroidetes ; Cytophagia ; Cytophagales ; Cytophagaceae ; Hymenobacter ; Unknown | 1.51 | 9.37 | 1.06 | 1.89 | 2.38 | 23.12 |
| Bacteria ; Bacteroidetes ; Cytophagia ; Cytophagales ; Cytophagaceae ; Hymenobacter ; Hymenobacter sp | 1.14 | 8.45 | 0.99 | 2.43 | 2.21 | 25.33 |
| Bacteria ; Cyanobacteria ; Unclassified ; Oscillatoriales ; Unclassified ; Unknown ; Unknown | 10.57 | 3.85 | 0.97 | 1.98 | 2.17 | 27.5 |
| Bacteria ; Proteobacteria ; Alphaproteobacteria ; Sphingomonadales ; Sphingomonadaceae ; Unknown ; Unknown | 16.52 | 16.82 | 0.94 | 1.28 | 2.1 | 29.6 |
| Bacteria ; Cyanobacteria ; Unclassified ; Nostocales ; Nostocaceae ; Nostoc ; Unknown | 5.68 | 8.97 | 0.94 | 1.67 | 2.09 | 31.69 |
| Bacteria ; Proteobacteria ; Alphaproteobacteria ; Rhodospirillales ; Unknown ; Unknown ; Unknown | 5.1 | 11.52 | 0.92 | 1.6 | 2.05 | 33.74 |
| Bacteria ; Actinobacteria ; Actinobacteria ; Pseudonocardiales ; Pseudonocardiaceae ; Pseudonocardia ; Pseudonocardia sp | 16.25 | 20.03 | 0.88 | 1.08 | 1.98 | 35.72 |
| Bacteria ; Proteobacteria ; Alphaproteobacteria ; Sphingomonadales ; Sphingomonadaceae ; Sphingomonas ; Sphingomonas sp | 16.73 | 19.75 | 0.88 | 1.11 | 1.97 | 37.69 |
| Bacteria ; Bacteroidetes ; Sphingobacteriia ; Sphingobacteriales ; Chitinophagaceae ; Unknown ; Unknown | 6.62 | 0.57 | 0.87 | 1.46 | 1.93 | 39.63 |
| Bacteria ; Cyanobacteria ; Unclassified ; Nostocales ; Nostocaceae ; Nostoc ; Nostoc sp | 6.23 | 11.96 | 0.85 | 1.94 | 1.89 | 41.52 |
| Bacteria ; Cyanobacteria ; Unclassified ; Nostocales ; Nostocaceae ; Unknown ; Unknown | 5.57 | 5.65 | 0.82 | 1.18 | 1.82 | 43.34 |
| Bacteria ; Cyanobacteria ; Unclassified ; Stigonematales ; Unclassified ; Loriellopsis ; Loriellopsis cavernicola | 5.4 | 0.78 | 0.76 | 1.11 | 1.69 | 45.03 |
| Bacteria ; Proteobacteria ; Alphaproteobacteria ; Rhodospirillales ; Acetobacteraceae ; Unknown ; Unknown | 11.27 | 7.41 | 0.76 | 1.81 | 1.69 | 46.72 |
| Bacteria ; Proteobacteria ; Alphaproteobacteria ; Rhizobiales ; Methylocystaceae ; Unknown ; Unknown | 7.32 | 1.92 | 0.74 | 2.7 | 1.66 | 48.38 |
| Bacteria ; Cyanobacteria ; Unclassified ; Nostocales ; Microchaetaceae ; Unknown ; Unknown | 6.91 | 5.19 | 0.72 | 1.13 | 1.6 | 49.98 |
| Bacteria ; Bacteroidetes ; Cytophagia ; Cytophagales ; Cytophagaceae ; Spirosoma ; Spirosoma sp | 2.64 | 5.79 | 0.71 | 1.18 | 1.59 | 51.57 |
| Bacteria ; Proteobacteria ; Alphaproteobacteria ; Sphingomonadales ; Unknown ; Unknown ; Unknown | 17.26 | 16.6 | 0.69 | 1.58 | 1.54 | 53.11 |
| Bacteria ; Bacteroidetes ; Cytophagia ; Cytophagales ; Cytophagaceae ; Spirosoma ; Spirosoma arcticum | 2.42 | 6.91 | 0.64 | 1.34 | 1.42 | 54.53 |
| Bacteria ; Acidobacteria ; Acidobacteriia ; Acidobacteriales ; Unclassified ; Unclassified ; Unclassified | 4.69 | 0.68 | 0.54 | 2.12 | 1.21 | 55.74 |
| Bacteria ; Actinobacteria ; Thermoleophilia ; Solirubrobacterales ; Solirubrobacteraceae ; Solirubrobacter ; Solirubrobacter sp | 5.36 | 1.71 | 0.5 | 1.49 | 1.12 | 56.86 |
| Bacteria ; Cyanobacteria ; Unclassified ; Oscillatoriales ; Unclassified ; Phormidesmis ; Phormidesmis priestleyi | 3.61 | 1.53 | 0.5 | 0.89 | 1.11 | 57.97 |
| Bacteria ; Proteobacteria ; Alphaproteobacteria ; Rhizobiales ; Hyphomicrobiaceae ; Rhodomicrobium ; Unknown | 4.05 | 0.64 | 0.47 | 1.5 | 1.06 | 59.03 |
| Bacteria ; Acidobacteria ; Acidobacteriia ; Acidobacteriales ; Acidobacteriaceae ; Acidobacterium ; Acidobacterium sp | 9.65 | 8.05 | 0.47 | 1.38 | 1.05 | 60.08 |
| Bacteria ; Actinobacteria ; Rubrobacteria ; Rubrobacterales ; Rubrobacteraceae ; Rubrobacter ; Rubrobacter sp | 3.75 | 2.48 | 0.46 | 1.27 | 1.02 | 61.1 |
| Bacteria ; Cyanobacteria ; Unclassified ; Oscillatoriales ; Unclassified ; Leptolyngbya ; Unknown | 5.26 | 2.85 | 0.46 | 1.41 | 1.02 | 62.11 |
| Bacteria ; Proteobacteria ; Alphaproteobacteria ; Rhodobacterales ; Rhodobacteraceae ; Unknown ; Unknown | 1.93 | 2.62 | 0.43 | 1.03 | 0.97 | 63.08 |
| Bacteria ; Proteobacteria ; Alphaproteobacteria ; Rhizobiales ; Unknown ; Unknown ; Unknown | 5.29 | 2.98 | 0.43 | 1.63 | 0.96 | 64.05 |
| Bacteria ; Proteobacteria ; Alphaproteobacteria ; Rhizobiales ; Methylobacteriaceae ; Methylobacterium ; Unknown | 5.42 | 2.95 | 0.41 | 1.49 | 0.91 | 64.96 |
| Bacteria ; Cyanobacteria ; Gloeobacteria ; Gloeobacterales ; Unclassified ; Gloeobacter ; Unknown | 3.68 | 0.8 | 0.4 | 1.53 | 0.89 | 65.85 |
| Bacteria ; Proteobacteria ; Alphaproteobacteria ; Rhodospirillales ; Acetobacteraceae ; Paracraurococcus ; Paracraurococcus ruber | 0.61 | 3.11 | 0.4 | 1.44 | 0.88 | 66.73 |
| Bacteria ; Bacteroidetes ; Cytophagia ; Cytophagales ; Cytophagaceae ; Spirosoma ; Unknown | 2.19 | 2.96 | 0.39 | 1.26 | 0.87 | 67.6 |
| Bacteria ; Proteobacteria ; Alphaproteobacteria ; Caulobacterales ; Caulobacteraceae ; Unknown ; Unknown | 3.21 | 1.18 | 0.36 | 1.55 | 0.8 | 68.4 |
| Bacteria ; Cyanobacteria ; Unclassified ; Pleurocapsales ; Unclassified ; Unclassified ; Unclassified | 2.73 | 0.33 | 0.33 | 2.29 | 0.75 | 69.15 |
| Bacteria ; Proteobacteria ; Alphaproteobacteria ; Rhizobiales ; Unclassified ; Unclassified ; Unclassified | 2.73 | 0.24 | 0.33 | 2.25 | 0.74 | 69.89 |
| Bacteria ; Bacteroidetes ; Sphingobacteriia ; Sphingobacteriales ; Sphingobacteriaceae ; Pedobacter ; Unknown | 3.35 | 1.6 | 0.32 | 1.47 | 0.72 | 70.61 |

| Supplementary Table 4. Indicator Eukaryotic species of Hypolith communities as determined by SIMPER analysis. Average Similarity=24.24 | | | | | |
| --- | --- | --- | --- | --- | --- |
| Species | Av.Abund | Av.Sim | Sim/SD | Contrib% | Cum.% |
| Plantae ; Chlorophyta ; Trebouxiophyceae ; Unclassified ; Coccomyxaceae ; Coccomyxa ; Coccomyxa subellipsoidea | 17.08 | 4.43 | 0.91 | 18.28 | 18.28 |
| Animalia ; Tardigrada ; Heterotardigrada ; Echiniscoidea ; Echiniscidae ; Unknown ; Unknown | 27.64 | 4.2 | 0.51 | 17.34 | 35.62 |
| Eukaryota ; Unclassified ; Spirotrichea ; Unknown ; Unknown ; Unknown ; Unknown | 13.32 | 3.9 | 1.12 | 16.1 | 51.72 |
| Plantae ; Chlorophyta ; Ulvophyceae ; Ulotrichales ; Unclassified ; Unknown ; Unknown | 6.63 | 1.67 | 0.86 | 6.89 | 58.61 |
| Plantae ; Chlorophyta ; Trebouxiophyceae ; Unclassified ; Unclassified ; Neocystis ; Neocystis brevis | 5.11 | 0.99 | 0.79 | 4.1 | 62.71 |
| Eukaryota ; Apicomplexa ; Coccidia ; Eucoccidiorida ; Eimeriidae ; Unclassified ; Unclassified | 3.64 | 0.99 | 1.18 | 4.08 | 66.79 |
| Plantae ; Chlorophyta ; Trebouxiophyceae ; Unclassified ; Unclassified ; Neocystis ; Unknown | 4.82 | 0.94 | 0.75 | 3.87 | 70.66 |

| Supplementary Table 5. Indicator Eukaryotic species of Epilith communities as determined by SIMPER analysis. Average Similarity=16.28 | | | | | |
| --- | --- | --- | --- | --- | --- |
| Species | Av.Abund | Av.Sim | Sim/SD | Contrib% | Cum.% |
| Plantae ; Chlorophyta ; Trebouxiophyceae ; Unclassified ; Coccomyxaceae ; Coccomyxa ; Coccomyxa subellipsoidea | 22.49 | 4 | 0.46 | 24.55 | 24.55 |
| Plantae ; Chlorophyta ; Trebouxiophyceae ; Chlorellales ; Chlorellaceae ; Chloroidium ; Chloroidium saccharophilum | 15.22 | 3.73 | 0.73 | 22.93 | 47.48 |
| Plantae ; Chlorophyta ; Ulvophyceae ; Trentepohliales ; Trentepohliaceae ; Trentepohlia ; Trentepohlia iolithus | 13.85 | 2.75 | 0.79 | 16.87 | 64.35 |
| Eukaryota ; Unclassified ; Colpodea ; Colpodida ; Unknown ; Unknown ; Unknown | 4.84 | 1.52 | 0.82 | 9.33 | 73.68 |

| Supplementary Table 6. Differentiator Eukaryotic species between Epilith and Hypolith communities as determined by SIMPER analysis. Average Dissimilarity=80.63 | | | | | |  |
| --- | --- | --- | --- | --- | --- | --- |
| Species | Av. Hypolith Abund | Av. Epilith  Abund | Av.Diss | Diss/SD | Contrib% | Cum.% |
| Animalia ; Tardigrada ; Heterotardigrada ; Echiniscoidea ; Echiniscidae ; Unknown ; Unknown | 27.64 | 8.5 | 9.46 | 1 | 11.73 | 11.73 |
| Plantae ; Chlorophyta ; Trebouxiophyceae ; Unclassified ; Coccomyxaceae ; Coccomyxa ; Coccomyxa subellipsoidea | 17.08 | 22.49 | 8.97 | 1.21 | 11.12 | 22.85 |
| Plantae ; Chlorophyta ; Trebouxiophyceae ; Chlorellales ; Chlorellaceae ; Chloroidium ; Chloroidium saccharophilum | 12.19 | 15.22 | 5.81 | 1.19 | 7.2 | 30.05 |
| Eukaryota ; Unclassified ; Spirotrichea ; Unknown ; Unknown ; Unknown ; Unknown | 13.32 | 7.14 | 5.48 | 0.97 | 6.8 | 36.86 |
| Plantae ; Streptophyta ; Jungermanniopsida ; Jungermanniales ; Unknown ; Unknown ; Unknown | 11.75 | 8.52 | 4.93 | 0.81 | 6.12 | 42.98 |
| Plantae ; Chlorophyta ; Ulvophyceae ; Trentepohliales ; Trentepohliaceae ; Trentepohlia ; Trentepohlia iolithus | 4.63 | 13.85 | 4.11 | 1.1 | 5.1 | 48.08 |
| Plantae ; Chlorophyta ; Ulvophyceae ; Ulotrichales ; Unclassified ; Unknown ; Unknown | 6.63 | 1.44 | 2.31 | 1.18 | 2.87 | 50.94 |
| Animalia ; Nematoda ; Chromadorea ; Tylenchida ; Aphelenchoididae ; Aphelenchoides ; Aphelenchoides sp | 3.63 | 0 | 1.94 | 0.44 | 2.4 | 53.34 |
| Animalia ; Nematoda ; Chromadorea ; Tylenchida ; Aphelenchoididae ; Aphelenchoides ; Unknown | 4.85 | 2.33 | 1.6 | 1.16 | 1.98 | 55.32 |
| Plantae ; Chlorophyta ; Trebouxiophyceae ; Unclassified ; Unclassified ; Neocystis ; Neocystis brevis | 5.11 | 0.44 | 1.54 | 1.24 | 1.91 | 57.24 |
| Plantae ; Chlorophyta ; Trebouxiophyceae ; Microthamniales ; Unclassified ; Elliptochloris ; Elliptochloris marina | 3.3 | 3.4 | 1.53 | 1.01 | 1.89 | 59.13 |
| Eukaryota ; Unclassified ; Colpodea ; Colpodida ; Unknown ; Unknown ; Unknown | 0.29 | 4.84 | 1.52 | 1.25 | 1.88 | 61.01 |
| Animalia ; Tardigrada ; Eutardigrada ; Parachela ; Hypsibiidae ; Ramazzottius ; Ramazzottius oberhaeuseri | 4.5 | 0.78 | 1.5 | 0.51 | 1.86 | 62.87 |
| Plantae ; Chlorophyta ; Trebouxiophyceae ; Unclassified ; Unclassified ; Neocystis ; Unknown | 4.82 | 1.59 | 1.46 | 1.27 | 1.81 | 64.68 |
| Plantae ; Chlorophyta ; Chlorophyceae ; Sphaeropleales ; Bracteacoccaceae ; Bracteacoccus ; Unknown | 4.81 | 0.5 | 1.42 | 1.09 | 1.76 | 66.44 |
| Eukaryota ; Unclassified ; Unclassified ; Cercomonadida ; Heteromitidae ; Unknown ; Unknown | 1.83 | 4.62 | 1.41 | 1.08 | 1.75 | 68.19 |
| Eukaryota ; Unclassified ; Dinophyceae ; Unknown ; Unknown ; Unknown ; Unknown | 3.01 | 1.3 | 1.33 | 0.92 | 1.65 | 69.85 |
| Fungi ; Ascomycota ; Dothideomycetes ; Capnodiales ; Mycosphaerellaceae ; Ramularia ; Ramularia grevilleana | 1.08 | 3.96 | 1.27 | 0.7 | 1.57 | 71.41 |
